# In silico analysis of prognostic and diagnostic significance of target genes from prostate cancer cell lines derived exomicroRNAs

**DOI:** 10.1186/s12935-023-03123-1

**Published:** 2023-11-17

**Authors:** Antonio Altuna-Coy, Xavier Ruiz-Plazas, Verónica Arreaza-Gil, José Segarra-Tomás, Matilde R. Chacón

**Affiliations:** 1 Disease Biomarkers and Molecular Mechanisms Group, IISPV, Joan XXIII University Hospital, Universitat Rovira I Virgili, C/Dr. Mallafré Guasch, 4,, 43007 Tarragona, Spain; 2 Urology Unit, Joan XXIII University Hospital, Tarragona, Spain

**Keywords:** Prostate cancer, Exovesicles, exomiRNAs, Diagnosis, Prognosis, Targets

## Abstract

**Background:**

Cancer-secreted exovesicles are important for cell-to-cell communication by altering cancer-related signalling pathways. Exovesicles-derived miRNAs (exomiRNAs)-target genes can be useful for diagnostic and prognostic purposes.

**Methods:**

ExomiRNA from prostate cancer (PCa) cells (PC-3 and LNCaP) were quantified by qRT-PCR and compared to the healthy cell line RWPE-1 by using miRNome PCR 752 miRNAs Panel. MiRNet database was used to predict exomiRNA-target genes. ExomiRNA-target genes pathway functional enrichment was performed by using Reactome database and Enrichr platform. Protein–protein interaction analysis was carried out by using the STRING database. RNA target-gene sequencing data from The Cancer Genome Atlas Prostate Adenocarcinoma (TCGA-PRAD) database was screened out in 465 PCa patients for candidate gene expression in prostate tumour (PT) tissue and non-pathologic prostate (N-PP) tissue. Signature gene candidates were statistically analysed for diagnosis and prognosis usefulness.

**Results:**

A total of 36 exomiRNAs were found downregulated when comparing PCa cells vs a healthy cell line; and when comparing PC-3 vs LNCaP, 14 miRNAs were found downregulated and 52 upregulated. Reactome pathway database revealed altered pathways and genes related to miRNA biosynthesis, miRNA-mediated gene silencing (*TNRC6B* and *AGO1*), and cell proliferation (*CDK6*), among others. Results showed that *TNRC6B* gene expression was up-regulated in PT tissue compared to N-PP (n = 52 paired samples) and could be useful for diagnostic purposes. Likewise, gene expression levels of *CDK6*, *TNRC6B,* and *AGO1* were down-regulated in high-risk PT (n = 293) compared to low-risk PCa tissue counterparts (n = 172). When gene expression levels of *CDK6*, *TNRC6B,* and *AGO1* were tested as a prognostic panel, the results showed that these improve the prognostic power of classical biomarkers.

**Conclusion:**

ExomiRNAs-targets genes, *TNRC6B*, *CDK6*, and *AGO1*, showed a deregulated expression profile in PCa tissue and could be useful for PCa diagnosis and prognosis.

**Supplementary Information:**

The online version contains supplementary material available at 10.1186/s12935-023-03123-1.

## Introduction

Prostate cancer (PCa) has become the most frequent cancer among Western men, accounting for a quarter of all types of tumours [1]. Overdiagnosis and subsequent overtreatment become a problem, and the search for biomarkers that could discriminate indolent localized PCa that can be followed by active surveillance from those aggressive localized PCa tumours that need radical treatment is necessary. Although clinical treatments such as surgery and endocrine therapy can effectively intervene in the progression of PCa, the prognosis of patients is often poor in the event of tumour metastasis or castration resistance [2]. Therefore, a better understanding of the molecular mechanism of PCa will contribute to early diagnosis and intervention. Thus, the study of communication between tumour cells can be crucial for gaining knowledge about tumour progression and development.

Prostate cell lines (cancer and non-cancer) established from patients have been crucial research models for gaining functional and mechanistic insight into the understanding of PCa pathobiology [3]. Within the tumour, cells can communicate with each other, by a direct interaction through membrane receptors or ligands, by releasing soluble molecules (growth factors, cytokines, and chemokines) or extracellular vesicles, also called exovesicles [4]. Extracellular vesicles affect not only the cellular microenvironment, but also distinct cells and tissues via circulation in biofluids [5]. Small exovesicles such as exosomes (< 200 nm) can exert an effect on neighbouring cells by participating in the creation of a specific microenvironment, particularly through their microRNAs (miRNAs) content.

MiRNAs are a class of endogenous, noncoding RNAs (19–22 nucleotides) that regulate post-transcriptional gene expression by binding to complementary seed sequences at the untranslated regions (UTRs) of their target mRNAs [6]. Mounting evidence suggests that aberrant miRNA expression contributes to the development of PCa [7]. Exovesicles released from the PCa cell lines have been found to contain miRNAs, indicating that these vesicles have the capacity to shuttle genetic information between cells [8]. Analysis of exovesicles-derived miRNAs has revealed insights into intercellular communication during invasion of different breast, prostate, and glioblastoma cancer cell lines, and several miRNAs were found to be significantly differentially abundant during cell invasion in the studied cell lines. Thus, a better understanding of miRNA profiles and their targets in the molecular carcinogenesis of PCa would be useful for early diagnosis and for designing therapeutic strategies.

Key miRNAs in PCa progression based on miRNA-mRNA interaction networks have been identified by a computational approach implicated in PCa onset and progression [9]. Thus, searching for specific miRNA-gene targets offers a novel approach for diagnostic and/or therapeutic strategies. For instance, epithelial-mesenchymal transition (EMT)-related genes such as Cadherin-2 (*CDH2*) or Vimentin (*VIM*) have been found to be regulated by exomiR-1246 in vitro in different PCa cells models [10]. TCF12—transcription factor 12 (*TCF12*) and Nemo Like Kinase (*NLK*) were found to be involved in PCa progression and to be regulated by exomiR-221-3p in PC-3 cells [11]. Though more novel targets are known to be important for PCa progression, their validation is still pending.

In the present study, we obtained differentially expressed exovesicle-derived miRNAs profiles from three different prostate cell lines: two tumoral, PC-3 (androgen-independent) and LNCaP (androgen-dependent), and one non-tumour epithelial prostate cell line, RWPE-1. Subsequently, the exomiRNAs-target genes were predicted, and enrichment pathway analysis was performed. After protein–protein interaction networks analysis, we downloaded the expression profile of candidate genes from *The Cancer Genome Atlas Prostate Adenocarcinoma* database (TGCA) and searched for expression in the PCa tissue samples database (PRAD). Finally, selected targets were evaluated for their usefulness for diagnostic and prognostic purposes.

## Materials & methods

### Prostate cancer cell lines culture conditions

Androgen-independent (PC-3), androgen-dependent (LNCaP) PCa cell lines and the non-tumour cell line, RWPE-1 (immortalized with papillomavirus 18) were purchased from Sigma-Aldrich (Barcelona, Spain). PC-3 cells were cultured in Ham’s F-12 K (Kaighn’s) Medium (1:1 mixture) with L-Glutamate (Gibco, Fisher Scientific SL, Madrid, Spain). LNCaP cells were cultured in RPMI 1640 medium (Gibco) supplemented with 1 mM Sodium Pyruvate (Gibco). Both PC-3 and LNCaP cells were supplemented with 10% foetal bovine serum (FBS) (FBS-depleted extracellular vesicles from Gibco). RWPE-1 cells were grown in keratinocyte serum-free medium (Gibco) plus 5 ng/ml bovine pituitary extract (BPE) and 50 μg/ml recombinant human epidermal growth factor (rhEGF). All cell line culture medium were supplemented with 1 × antibiotic–antimycotic (Gibco) . Cells were grown in a humidified cell culture incubator (5% CO_2_ atmosphere at 37 ℃). All cell lines were tested for mycoplasma contamination and kept free of mycoplasma by adding 5 μg/ml Plasmocin^TM^ (InvivoGen, San Diego, CA, USA) to the culture media.

### Transmission electron microscopy extracellular vesicles identification

Extracellular Vesicles from 16 ml of culture cell medium were isolated using a modified procedure from the exoRNeasy Serum/Plasma Maxi Kit (Qiagen, BioNova Cientifica, Madrid, Spain), by eluting extracellular vesicles in 500 μl of Buffer XE (Qiagen) before extracting miRNA-cargo. Eluates containing intact extracellular vesicles were concentrated using a 100,000 Da cut-off concentrator Amicon Ultra-2 ml Centrifugal Filters (Millipore, Fisher Scientific SL, Madrid, Spain). Then, concentrated isolated extracellular vesicles were diluted 2:5 with Buffer XE (Qiagen), placed on carbon-coated copper grids (200 mesh), and incubated in osmium tetroxide vapor for 15 min. Images were collected using a JEOL 1011 transmission electron microscope (Jeol, Tokyo, Japan) operating at 80 kV with a megaview III camera (Olympus Soft Imaging Solutions GmbH, Munster, Germany).

### ExomiRNAs isolation from PCa cell culture

A volume of 16 ml of culture media was collected after 24 h incubation and filtered with Minisart^®^ syringe 0.8 μm filters (Sartorious, Fisher Scientific SL, Madrid, Spain). ExomiRNAs were isolated using exoRNEasy Serum/Plasma Maxi Kit (Qiagen) following the manufacturer's instructions.

### ExomiRNAs expression analysis and screening

For exomiRNAs expression analysis, reverse transcription was undergone using miRCURY LNA Universal RT microRNA PCR, Polyadenylation, and cDNA Synthesis Kit (Qiagen). cDNA was diluted and assayed by qRT-PCR on a 7900HT Fast Real-Time PCR System (Applied Biosystems, Foster City, CA, USA) using ExiLENT SYBR Green Master Mix on the Human panel I + II, V5, miRCURY LNA miRNA miRNome PCR Panel (Qiagen) that included 752 mature human cancer-related miRNAs following the manufacturer’s instructions. Fluorescence readings and expression recordings of exomiRNAs during qRT-PCR were performed with Design & Analysis (DA2) program (Applied Biosystems). From the quantitative qRT-PCR analysis of all miRNAs analysed, only miRNAs showing C_T_ expression levels < 33 were considered. GeneGlobe software (https://geneglobe.qiagen.com/us/analyze) (Qiagen) was used and C_T_ values for each sample were normalized using arithmetic mean of the expression values of the following miRNAs: hsa-miR-423-5p, SNORD38B, SNORD49A, hsa-miR-191-5p, hsa-miR-103a-3p and U6 small nuclear RNA. The fold change expression was calculated with the formula 2^−∆∆Ct^. ExomiRNAs with fold change ≥ 1.8 or ≤ − 1.8 were considered for further analysis.

### ExomiRNAs-target and pathway functional enrichment analysis

To predict potential exomiRNA targets, miRNet platform (https://www.mirnet.ca) was used. Potentially functional altered pathways were analysed with Reactome (https://reactome.org) using Enrichr (https://maayanlab.cloud/Enrichr/) to generate enrichment maps.

### RNA target-gene sequencing bioinformatic data analysis

RNA-sequencing expression profiles of 465 prostate tumour (PT) tissue and 52 non-pathologic prostate (N-PP) tissue from *The Cancer Genome Atlas Prostate Adenocarcinoma prostate cancer* database (TCGA-PRAD) project were downloaded from the CancerMIRNome resource (http://bioinfo.jialab-ucr.org/CancerMIRNome/) to analyse target-gene exomiRNAs expression. Clinical data of TCGA PRAD subjects was also downloaded from GDC Data Portal (https://portal.gdc.cancer.gov/). The deregulated exomiRNA-target interactions were individually validated using the miRWalk platform (http://mirwalk.uni-hd.de/). The calculated score was generated by executing TarmiR algorithm for miRNA target site prediction. The closer the score to 1, the greater the confidence prediction [12].

### Statistical analysis

The parametric T-Student’s test was used to analyse relative exomiRNAs expression levels between cell lines on the Geneglobe website program. The non-parametric Mann–Whitney U-test was used to analyse the differences in the relative expression levels of the candidate’s target genes between studied groups and Kruskal Wallis-test was used to analyse any differences between ISUP groups. A p-value of less than 0.05 was considered statistically significant. Univariate binary logistic regression models and receiver operating characteristic (ROC) analysis were performed to evaluate the best predictive model. Classification and Regression Trees (CART) analysis was performed to determine the performance of the best predictive model. The SPSS Statistics 26.0 statistical package (IBM, Madrid, Spain) and R software were used for analysis. Graph Pad Prism V9 was used to plot data results.

## Results

### Isolation and characterization of extracellular vesicles from PCa cell medium

**Fig. 1:**
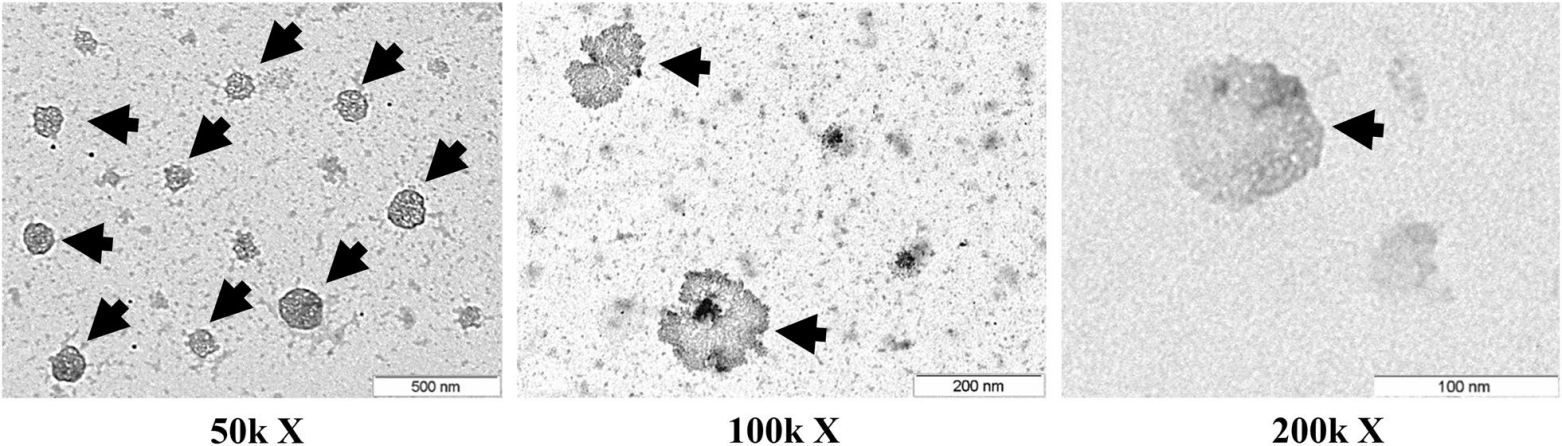
Characterization of isolated extracellular vesicles. Analysis of extracellular vesicles (EVs) by electron microscopy at different magnifications. Arrows indicate osmium tetroxide deposition on the extracellular vesicle’s lipid surface

Isolated extracellular vesicles purity and size were tested by transmission electron microscopy (TEM) from prostate cells culture medium treated with osmium tetroxide to stain lipid-bilayer [13]. Osmium TEM staining images revealed that the observed dense bodies raged in size from 50 to 150 nm, corresponding to a small extracellular vesicle size range, a group that includes exosomes [4] (Fig. 1). Following the International Society of Extracellular Vesicles (ISEV) guidelines, [4] we previously verified that PCa cell-derived exovesicles were enriched in CD9, CD81, and HSP70 and published [11].

### Analysis of exomiRNAs cargo between prostate cancer epithelial cell lines (PC-3 and LNCaP) and healthy epithelial cell line (RWPE-1) reveals a small number of shared deregulated exomiRNAs profile patterns

**Fig. 2:**
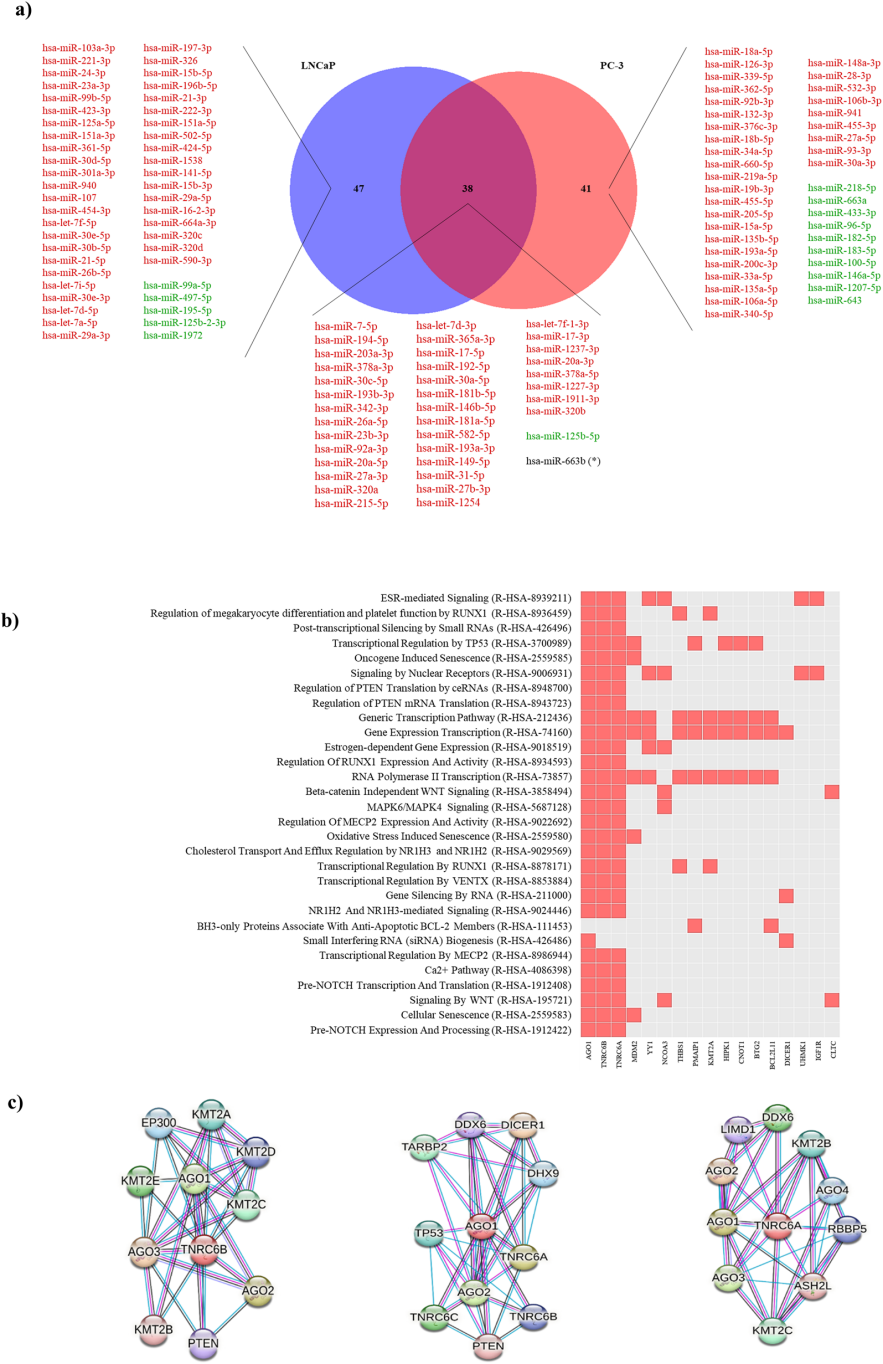
Gene set enrichment and exomiRNAs target-gene analysis of deregulated exo-miRNAs in PCa cells vs RWPE-1 cell line. **a** ExomiRNAs differentially expressed in PCa cell lines (PC-3 and LNCaP) versus healthy cell line (RWPE-1). The Venn diagram shows the number of exomiRNAs differentially expressed in LNCaP (blue circle), PC-3 (red circle) and in both regarding the normal healthy prostate epithelial cell line (RWPE-1) (purple circle). For each category, the exomiRNAs upregulated (indicated in green) and downregulated (indicated in red) are indicated in the diagram. (*) hsa-miR-663b is overexpressed in PC-3 and under-expressed in LNCaP relative to RWPE-1. **b** Reactome pathway enrichment analysis list of significant pathways affected by downregulated exomiRNAs in PCa cells and putative implicated target genes. **C** STRING analysis of protein–protein interaction network of the exomiRNAs target genes TNRC6B, AGO1 and TNRC6A. Coloured circle predicted functional protein partners. Different colours lines represent different evidence of a connection between proteins (Black—Co-expression, pink—experimental evidence, turquoise—database evidence)

The Venn-Diagram represents the differentially expressed exomiRNAs between prostate cancer cell lines (PC-3 and LNCaP) and the healthy control cell line (RWPE-1) (Fig. 2a). A total of 47 exomiRNAs were differentially expressed when comparing LNCaP vs RWPE-1, while 41 exomiRNAs were found to be deregulated between PC-3 and RWPE-1 (Additional file 1). When the deregulated exomiRNAs of PCa cells (PC-3 and LNCaP) were compared to those of RWPE-1, 36 exomiRNAs were found to be downregulated, and only one, hsa-miR-125b-5p was upregulated. In contrast, hsa-miR-663b was found overexpressed in PC-3 cells and down-regulated in LNCaP cells (Fig. 2a). Interestingly, 626/ 752 (83%) exomiRNAs analysed were equally expressed in all 3 cell lines.

### Trinucleotide repeat-containing gene 6B (*TNRC6B)* expression can be a candidate gene useful for PCa diagnosis.

We studied in silico the impact of the 36 downregulated PCa cells exomiRNAs on target genes. Using miRNet analysis, potential interactions between these downregulated exomiRNAs and their target genes showed 41 genes potentially regulated by 21/36 exomiRNAs (Additional file 5: Figure S1a). Then, we searched for pathways regulated by these 41 genes. For this, Reactome enrichment analysis database was used (Fig. 2b) and the result revealed 30 signalling pathways related to classical mitogen-activated protein kinase (MAPK), Wnt/β-Catenin signalling pathway (WNT), nuclear receptors signalling, gene transcription, translation, and gene silencing among others; and were found to share 3 target genes (Fig. 2b, c): Trinucleotide repeat-containing gene 6B (*TNRC6B*), Argonaute RISC Component 1 (*AGO1*) and Trinucleotide repeat-containing gene 6A (*TNRC6A*). Full results of Reactome Pathway enrichment are provided in Supplementary Material (Additional file 2).

Then, we conducted a protein–protein interaction analysis with STRING database and results showed that putative proteins related with the TNRC6B, AGO1, and TNRC6A proteins were mainly related with miRNA biosynthesis (DICER1), with genes involved in miRNA-mediated gene silencing (Argonaute RISC Component 2 (AGO2), Argonaute RISC Component 3 (AGO3), Argonaute RISC Component 4 (AGO4), LIM domain containing 1 (LIMD1), DEAD-Box Helicase 6 (DDX6) histone-lysine N-methyltransferase 2-dependent epigenetic-regulatory mechanism (KMT2A, KMT2B, KMT2C, KMT2D, and KMT2E) or with tumour suppressor genes such TP53 and Phosphatase and tensin homolog (PTEN) among others (Fig. 2c).

**Fig. 3:**
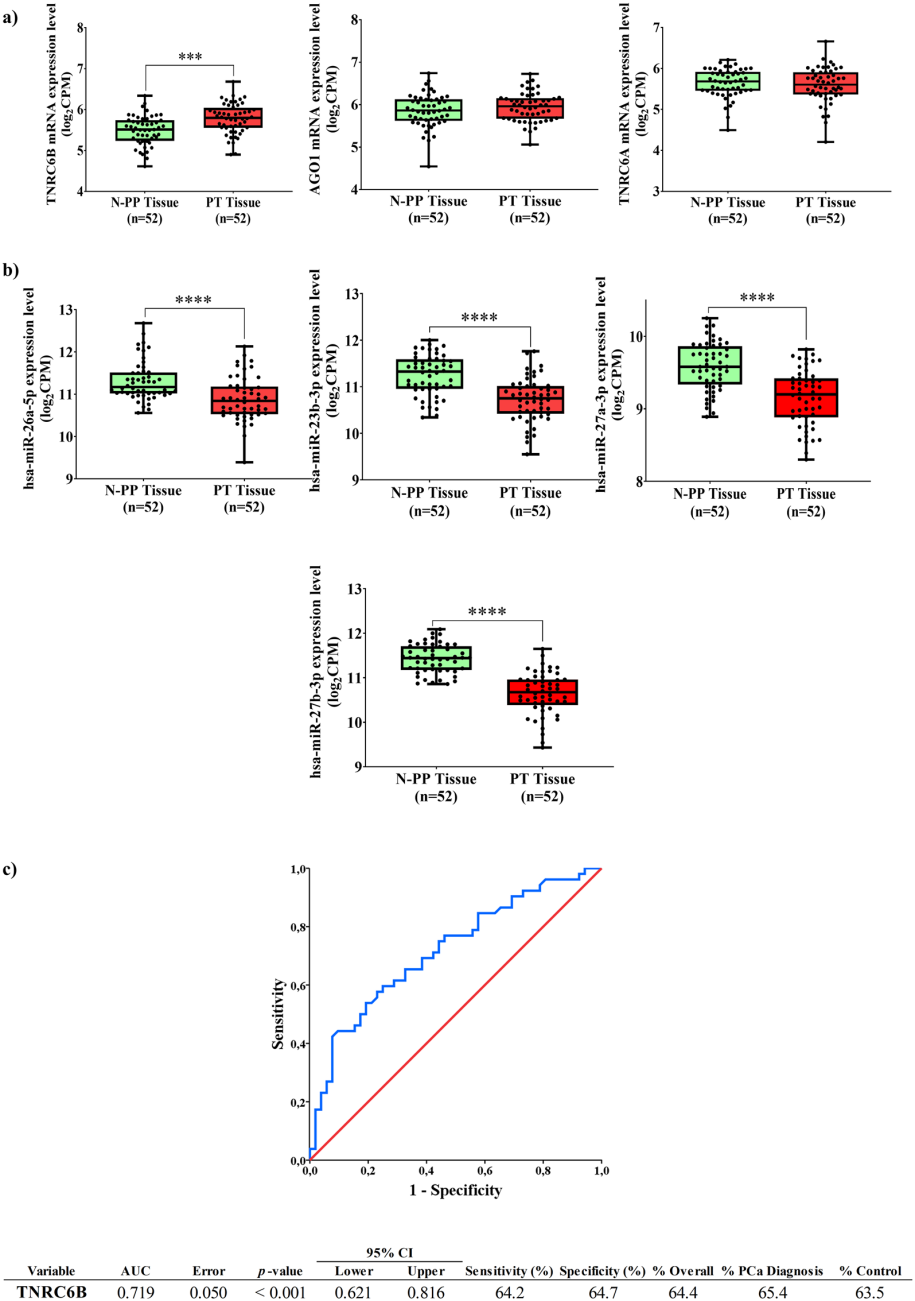
ExomiRNAs-associated genes useful for PCa diagnosis. **a** Boxplot showing mRNA expressions of *TNRC6B*, *AGO1,* and *TNRC6A* genes in paired samples of Non-Pathologic Prostate (N-PP) Tissue and Prostate Tumour (PT) Tissue; obtained from The Cancer Genome Atlas Prostate Adenocarcinoma database (TCGA-PRAD). Box plots show the median, quartiles, and extreme values. ***p < 0.001. **b** Boxplot showing *TNRC6B* gene-associated exomiRNAs expression levels in paired samples of Non-Pathologic Prostate (N-PP) Tissue and Prostate Tumour (PT). ****p < 0.0001. **c** Receiver operating characteristic (ROC) curve values showing the predictive efficiency of *TNRC6B* gene expression in distinguishing N-PP from PT tissue. AUC, area under the curve; 95% CI (confidence interval)

To determine the expression of selected genes in PCa human tumours, data from 52 PT Tissue and N-PP from TCGA-PRAD database were analysed. Clinical data from the patients are listed in Additional file 6: Table S1. The expression pattern of the three genes revealed that *TNRC6B* expression was significantly up-regulated in PT Tissue compared to its paired N-PP (Fig. 3a). The expression of *AGO1* and *TNRC6A* was similar between N-PP and PT Tissue (Fig. 3a).

Correlations between the 21 differentially expressed exomiRNAs and *TNRC6B* gene expression were tested by Spearman’s bivariate correlation test (Additional file 5: Figure S2a). The most relevant associations observed were significant negative correlations between *TNRC6B* gene expression and the expression levels of hsa-miR-26a-5p, hsa-miR-23b-3p, hsa-miR-27a-3p and hsa-miR-27b-3p. Analysis of the expression behaviour of the 4 selected exomiRNAs in PT Tissue and N-PP is shown in Fig. 3b and all above-mentioned exomiRNAs showed a significantly decreased expression levels compared to the N-PP tissue expression.

We then checked using the miRWalk web server, the binding sites prediction for the 4 selected exomicroRNA. Sites were detected for 3/4 exomiRNAs in all 3 isoforms of the *TNRC6B* gene **(**Additional file 6: Table S2).

Then, to evaluate the usefulness of *TNRC6B* expression as a potential diagnostic biomarker of PCa, we performed a binary logistic regression and ROC curve analysis using the information from 52 tissue samples from TCGA-PRAD. The results showed that the area under the curve (AUC) of each individual variable was 0.719 (95% Confidence Interval: 0.621–0.816), with 64.7% specificity and 64.2% sensitivity (Fig. 3c) and was able to identify with certainty 65% of PCa patients.

### Differential exomiRNAs cargo profile between PC-3 and LNCaP reveals deregulated miRNAs -target genes involved in tumour proliferation and miRNA biogenesis

**Fig. 4:**
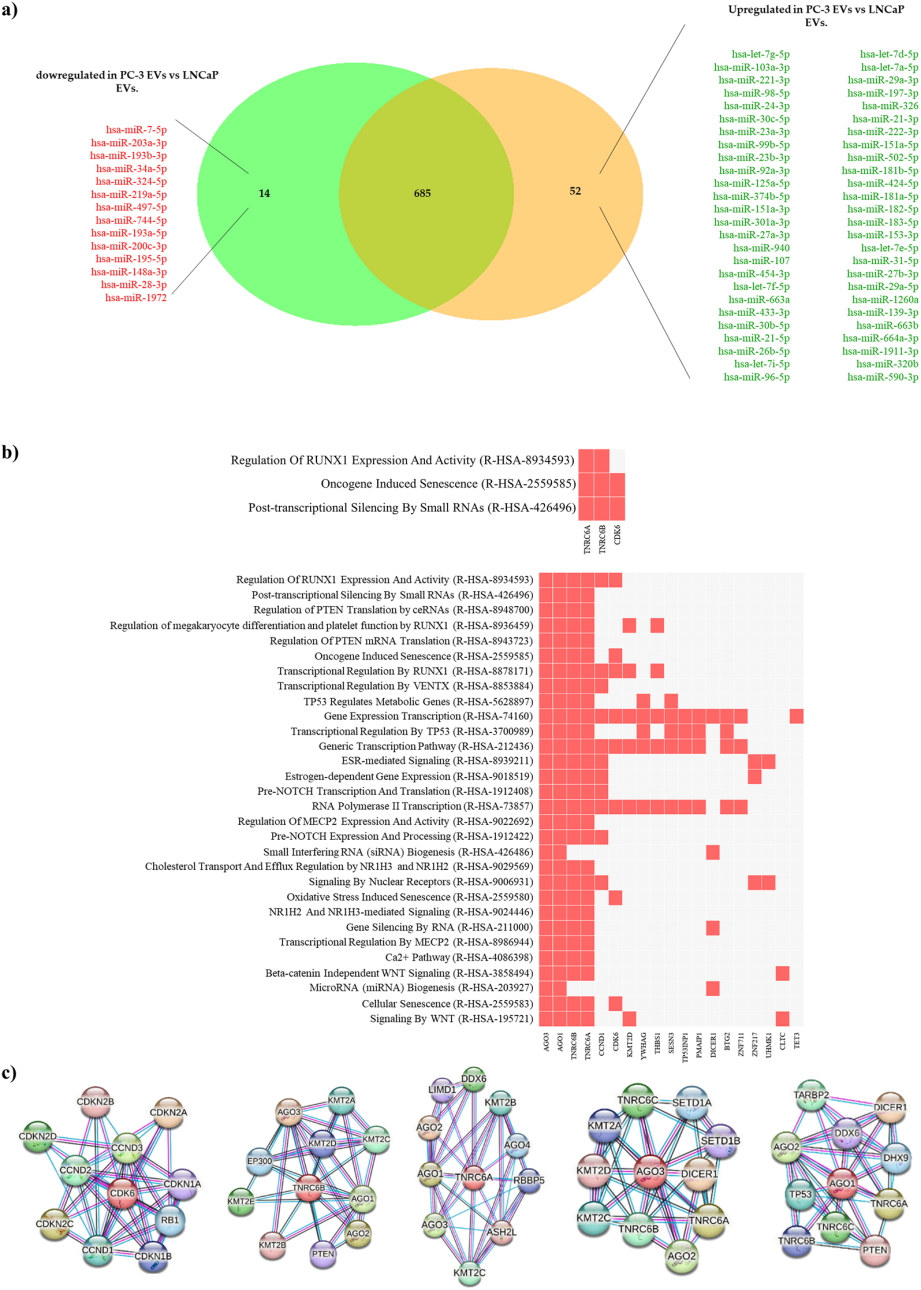
Gene set enrichment and exomiRNAs target-gene analysis of deregulated exo-miRNAs between PC-3 and LNCaP cell lines. **a** ExomiRNAs differentially expressed between PC-3 and LNCaP cell lines. The Venn diagram shows the number exomiRNAs under-expressed (green circle) and overexpressed (orange circle) in PC-3 regarding LNCaP. The yellow area represents exomiRNAs that are equally expressed between both cell lines. **b** Reactome pathway enrichment analysis list of significant pathways affected by deregulated exomiRNAs in PC-3 cells vs LNCaP cell lines and putative implicated target genes. **c** STRING analysis of protein–protein interaction network of exomiRNAs target genes CDK6, TNRC6B, AGO1, AGO3 and TNRC6A. Coloured circle predicted functional protein partners. Different colours lines represent different evidence of connection between proteins (Black—Co-expression, pink—experimental evidence, turquoise—database evidence)

The Venn-Diagram in Fig. 4a shows the exomiRNAs differentially deregulated in PC-3 vs LNCaP. Of the total 752 exomiRNAs analysed, 14 exomiRNAs were found to be downregulated in PC-3 compared to LNCaP, while 52 exomiRNAs were found to be upregulated (Additional file 1). Interestingly, 685 of the 752 (91%) exomiRNAs were equally expressed in both cell lines.

We then studied in silico the genetic impact of the 14 downregulated and 52 upregulated exomiRNAs on putative target genes. By using miRNet, potential interactions between downregulated exomiRNAs and their target genes revealed that 38 genes could potentially be regulated by at least 8/14 downregulated exomiRNAs (Additional file 5: Figure S1b). Reactome pathway database and *Enrichr* were used to obtain the pathways regulated by these 38 genes. The results showed shared significant pathways regulated by miRNA-targeted genes such as the Runt-related transcription factor 1 (*RUNX1*) pathway, post-transcriptional silencing of small RNAs, and oncogene-induced senescence pathway (Fig. 4b). Full results of pathway-based reactome enrichment analysis are included in Additional file 3.

When miRNA-gene interaction analysis was performed for the upregulated exomiRNAs using miRNet software, 43 genes were found to be potentially regulated by at least 29/52 exomiRNAs (Additional file 5: Figure S1c). Reactome pathway enrichment analysis revealed that RUNX1 signalling, oncogenes induced senescence were also under the control of these miRNAs. Cancer miRNA biogenesis, cell cycle, and the PI3K-Akt signalling were among the pathways regulated by exomiRNAs (Fig. 4b). Full results of Reactome Pathway enrichment are provided in Additional file 4.

Protein–protein interaction analysis was performed on the STRING database, which included cell division protein kinase 6 (CDK6), trinucleotide repeat containing adaptor 6B (TNRC6B), argonaute RISC component 1 (AGO1), argonaute RISC component 3 (AGO3) and trinucleotide repeat containing adaptor 6A (TNRC6A). The results showed that CDK6 is related to cell proliferation regulatory proteins such as cyclin-dependent kinase 4 inhibitor A (CDKN2A), cyclin-dependent kinase 4 inhibitor B (CDKN2B), or the cyclin-dependent kinase inhibitor 1 (CDKN1A or p21) among others and the tumour suppressor protein retinoblastoma (RB1) (Fig. 4c). Furthermore, TNRC6B, TNRC6A, AGO1, and AGO3 putative proteins are mainly related to miRNA biosynthesis and miRNA-mediated gene silencing (DICER1 or AGO2 among others), epigenetic-regulatory proteins (Histone-lysine N-methyltransferase 2A, B, C and D (KMT2A, KMT2B, KMT2C, and KMT2D)), proliferative genes (SET domain containing 1A and 1B (SETD1A and SETD1B) and tumour suppressor genes such TP53 and PTEN among others (Fig. 4c).

### The expression of *CDK6*, *TNRC6B,* and *AGO1* in prostate tumour tissue is related to PCa aggressiveness and could be used as a panel to improve PCa prognosis

**Fig. 5:**
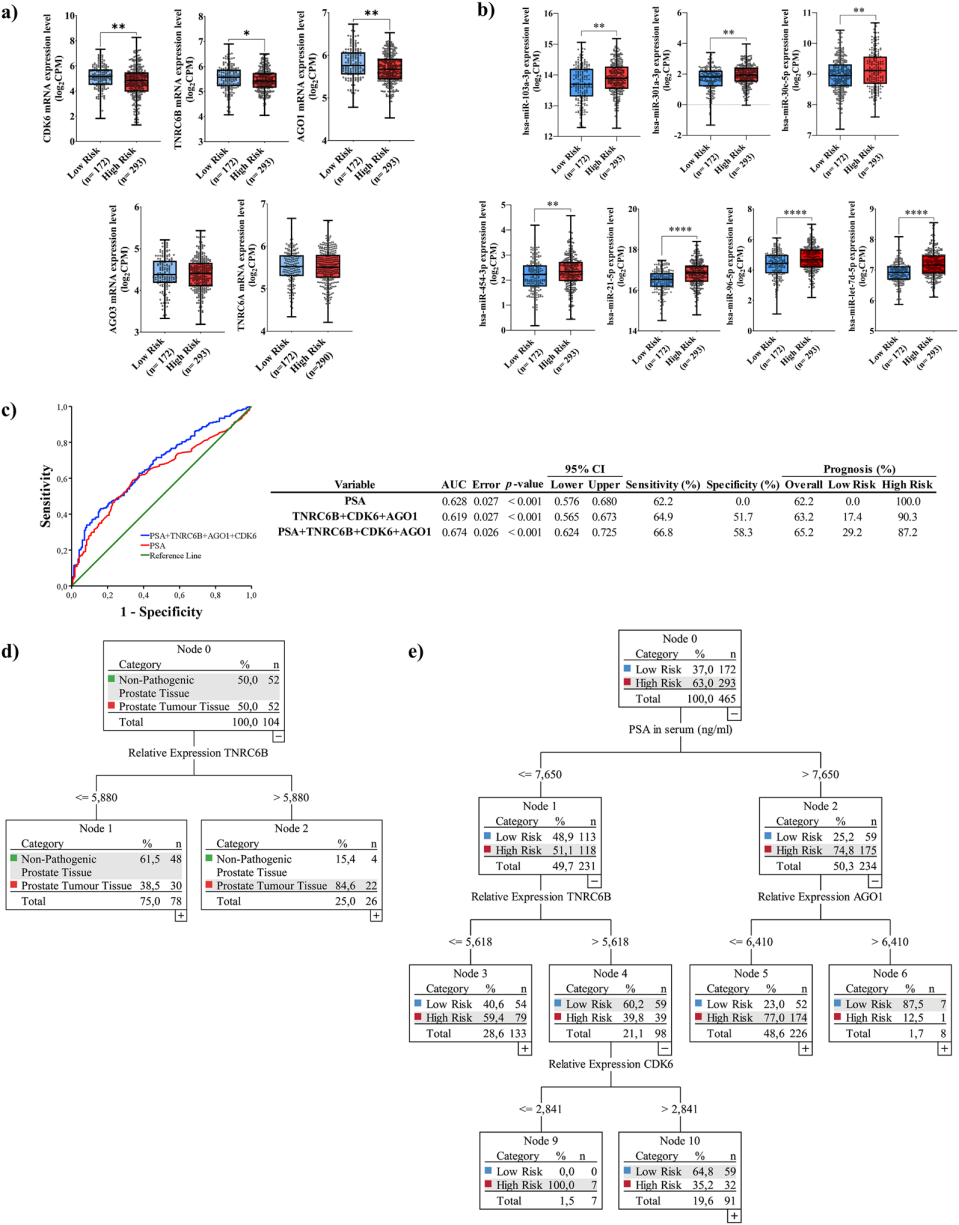
ExomiRNAs-associated genes are useful for PCa prognosis. **a** Boxplot showing mRNAs gene expression of *CDK6, TNRC6B*, *AGO1*, *AGO3,* and *TNRC6A* in prostate tumour tissue samples of patients stratified according to ISUP-GG classification (Low Risk: ISUP I and II; High Risk: ISUP III, IV, and V) from TCGA-PRAD database. Boxplots show median, quartiles, and extreme values. *p < 0.05, **p < 0.01. **b** Boxplot showing deregulated-exomiRNAs in prostate tumour tissue samples of patients stratified according to ISUP-GG classification (Low Risk: ISUP I and II; High Risk: ISUP III, IV, and V) from the TCGA-PRAD database. Boxplots show median, quartiles, and extreme values. **p < 0.01. ****p < 0.0001. **c** Receiver operating characteristic (ROC) curve values showing the predictive efficiency for prostate cancer aggressiveness of selected genes compared to PSA. AUC, area under the curve; 95% CI (confidence interval). **d** Classification And Regression Decision Tree (CART) model for PCa diagnosis based on TNRC6B expression levels in PT-Tissue. **e** Classification And Regression Decision Tree (CART) model for PCa prognosis. The decision tree was built up with the following parameters: PSA levels in serum and the gene expression of *CDK6*, *TNRC6B,* and *AGO1* PT-Tissue

We checked the expression levels of *CDK6*, *TNRC6B, AGO1*, *AGO3,* and *TNRC6A* in PCa tissues, based on data from 465 prostate tumour samples obtained from TCGA-PRAD database, and stratified according to the 2014 ISUP-GG [14] into low-risk (ISUP I and II) and high-risk (ISUP III, IV, and V). Clinical data of patients are listed in Additional file 6: Table S3. Gene expression pattern analysis of *CDK6*, *TNRC6B,* and *AGO1* was significantly decreased in high-risk PCa patients’ samples compared to their lower-risk counterparts (Fig. 5a), whereas the expression of *AGO3* and *TNRC6A* genes showed no significant differences between low and high-risk groups (Fig. 5a). Interestingly, when we plotted the expression of the above-mentioned genes according to ISUP, we observed a significant difference in the expression levels of *CDK6, TNRC6B*, and *AGO1* in patients with ISUP V with respect to patients with ISUP II (Additional file 5: Figure S3a).

When *CDK6*, *TNRC6B,* and *AGO1* gene expression levels obtained from the TCGA-PRAD database were plotted regarding tumour aggressivity attending to lymph node invasion, N0 and N1, we observed a decreased expression in TNRC6B did not reach significance (Additional file 5: Figure S3b).

Moreover, spearman’s bivariate correlations between the 29 differentially expressed exomiRNAs and *CDK6*, *TNRC6B,* and *AGO1* gene expression were performed (Additional file 5: Figure S2b). The most relevant exomiRNAs-gene associations observed were the significant negative correlations between *CDK6*, *TNRC6B,* and *AGO1* gene expression and the expression levels of hsa-miR-103a-3p, has-miR-30c-5p, has-miR-301a-3p, has-miR-454-3p, has-miR-21-5p, has-miR-96-5p and has-let-7d-5p. Analysis of the expression behaviour of the 7 selected exomiRNAs in 465 prostate tumour samples, stratified according to aggressiveness is shown in Fig. 5b. In addition, predicted target-miRNA binding sites were detected for 6/7 selected exomicroRNAs, using miRWalk web server **(**Additional file 6: Table S2).

To evaluate the usefulness of CDK6, TNRC6B, and AGO1 expression in prostate tumour tissue as potential prognosis biomarkers of PCa aggressiveness, we performed a ROC curve analysis of the above-mentioned gene expressions alone and in conjunction with serum PSA levels. The results showed that the panel composed of 4 variables, serum PSA levels and the expression of CDK6, TNRC6B, and AGO1, has an AUC of 0.674 (95% Confidence Interval 0.624–0.725), with 58.3% specificity and 66.8% sensitivity, improving the prognosis power of serum PSA levels, as well as the prognosis power of only the expression of these genes (Fig. 5c**)**.

### Cut-off gene expression values for PCa clinical stratification by using candidate-selected miRNA-target genes

An analysis was performed using the Classification and Regression Decision Tree (CART) and the best predictive selected genes were obtained by binary logistic regression and ROC. Results show that 42.3% (22/52) of subjects with TNRC6B tissue expression levels > 5.88 can be correctly classified as PCa (Fig. 5d).

While for prognosis by using serum PSA levels and *CDK6, TNRC6B,* and *AGO1* expression in prostate tumour tissue, the CART chart showed that a patient with PSA values ≤ 7.65 ng/ml, will harbour a high-risk PCa if the relative expression level of *TNRC6B* in PCa tissue is ≤ 5.618 (27%). But if the relative expression of *TNRC6B* is > 5.618 and the relative expression of *CDK6* is ≤ 2.841, patients can be classified as high-risk PCa, concretely, 2.4% of subjects can be correctly advanced the presence of an aggressive tumour (Fig. 5e). But if a patient has PSA values > 7.65, and the relative expression of *AGO*1 is ≤ 6.41, 59.4% of patients can be classified as potentially having an aggressive tumour (Fig. 5e). Thus, the overall correct prognosis performance is 66/172 (38.4%) as a low risk and 260/293 (88.7%) as a high-risk patient (Fig. 5e).

## Discussion

In the search to understand the complex communication in the tumour microenvironment by exomiRNAs derived from exovesicles, we used PCa cell lines to provide information on tumour development and progression without the limitation of tissue availability. Regarding PCa development, exomiRNA cargo between PCa epithelial cell lines (PC-3 and LNCaP) and healthy epithelial cell line (RWPE-1) was undertaken revealing that only 17% (126/752) of the exomiRNAs were deregulated, while 83% (626/752) were shared. These data agree with previous reports comparing the exomiRNA expression pattern in PC-3 with RWPE-1 cells [8].

In silico tools exhibit three shared miRNA target genes implicated among the most significantly potentially dysregulated pathways: Trinucleotide repeat-containing gene 6B *and 6A (TNRC6B* and *TNRC6A)* and argonaute RISC component 1 (*AGO1)*. According to the data from microarray analysis on 52 paired PCa samples from PCa tumour area and non-pathological tissue obtained from TCGA, we observed that relative expression of the *TNRC6B* gene was significantly upregulated in prostate tumour tissue. Accumulative evidence demonstrates that *TNRC6B* is involved in RNA-mediated gene silencing by both miRNAs and short-interfering RNAs (siRNAs) [15–17]. A probabilistic approach identified the *TNRC6B* gene as a competing endogenous RNA (ceRNA) of phosphatase and Tensin gene (*PTEN)* in PCa [18] contributing to the suppression of the expression of PTEN, a common event of PCa development [19, 20]. Furthermore, single nucleotide polymorphisms (SNPs) variants in the *TNRC6B* gene have been associated with PCa risk [21, 22]. Our results indicate that at least 3/4 downregulated exomiRNAs from prostate tumour tissue may positively regulate the expression of the *TNRC6B* gene because binding sites were predicted, a fact that may involve this gene in the impairing of multiple signalling pathways linked to PCa development. Since the prognostic value had not been evaluated in the context of prostate cancer to date, we performed binary logistic regression and ROC curve analysis. The results obtained showed that *TNRC6B* gene expression had a diagnostic accuracy of PCa of 65%, which is quite acceptable considering we are using a single biomarker; although we were unable to compare the diagnostic power obtained for *TNRC6B* gene expression levels with the diagnostic power of classic PSA biomarker used for PCa diagnosis because gene expression levels were retrieved from the same patient but from different tissue site, PT and N-PP, it still exceeds PSA serum diagnostic values as stated in the literature[23].

We also used exovesicles-derived exomiRNAs from PCa cell lines to search for information on PCa tumour progression. Only 8% (66/752) of exomiRNAs were differentially expressed in PC-3 compared to LNCaP cells. Using the same in silico procedure analysis described above, we found that potentially deregulated pathways were mostly related to tumour aggressiveness. Five target genes were found to be involved among the significantly dysregulated pathways: *TNRC6B*, *TNRC6A*, *AGO1*, *AGO3,* and *CDK6*. Both, downregulated and upregulated exomiRNA were found to target these genes, stressing their crucial role in PCa cellular processes. From the TCGA-PRAD database, we downloaded information on PCa patients according to ISUP-GG that were clustered into low-risk (ISUP I and II) and high-risk (ISUP III, IV, and V) groups. By doing so, we found a significant decrease in the expression of *TNRC6B, AGO1,* and *CDK6* between the low-risk and high-risk groups, pointing to a decrease in the repressive machinery of microRNAs and proliferation in the aggressive tumours. Moreover, when we checked the expression of tumour lymph node involvement, N0 and N1, we observed a lower expression of *TNRC6B* in the N1 group, indicating that decrease expression may be related to tumour aggressivity. In line with this finding, the *TNRC6B* expression was also found to be suppressed in hormone-refractory metastatic PCa compared to prostate carcinoma (http://www.oncomine.org).

CDK6 is a key player in the cell cycle G1/S phase transition along with CDK4, and its aberrant activity and gene expression have been found to be associated with tumour progression in multiple human cancers [24]. A recent study demonstrated that CDK6 is upregulated in PCa and could be a potential therapeutic target [25] as it was also described in other cancers such as bladder, melanoma, or pancreas [26, 27]. Moreover, CDK6 can promote PCa progression stimulating AR activity [28], and CDK4/6 inhibitors (combined olaparib and palbociblib treatment or abemaciclib) have been shown to induce apoptosis in PCa cell lines [29]. AGO1 plays a crucial role in the regulation of gene expression through the RNA-induced silencing complex (RISC) pathway [30]. In PCa cell lines, it has been demonstrated that AGO1 enhances the recruitment of RNA Polymerase II for cell growth and survival-related promoters’ genes [31].

In view of these findings, we evaluated by performing binary logistic regression and ROC the prognostic power of *CDK6, TNRC6B,* and *AGO1* to improve the prognostic power of serum PSA. The results confirmed the lack of specificity of PSA for PCa, documented in previous studies [23, 32]. Moreover, the lack of specificity of PSA is also confirmed after performing the prognostic CART chart since all patients are classified as high risk. After performing several variable combinations, the panel composed of CDK*6, TNRC6B, AGO1,* and PSA was shown to be the best combination, improving the specificity (58%) and sensitivity (4.6%) over PSA. Thus, this panel may help to detect aggressive PCa tumours better than PSA.

The present study has certain limitations. We are aware that working with in vitro prostate cancer cell line models does not reflect the entire complexity of the biological systems simulated. Immortalized PCa cell lines originating from the prostatic tissue of PCa patients with localized disease are not accessible to the research community; thus, we used PCa cell lines that exhibit gene expression patterns like a human tumour. Additionally, as target predictions are getting better and, as the number of data deposited on cancer databases is increasing, this type of analysis represents a great opportunity for clinical research by increasing the number of samples. Despite that, we know that the results presented here should be used with caution, as their validation is still pending.

## Conclusion

In the present study, we revealed 4 deregulated PCa cell exomiRNAs can epigenetically control the *TNRC6B* gene, showing to be useful for PCa diagnosis; whereas *TNRC6B*, *CDK6*, and *AGO1* were found to be potentially regulated by 7 PCa cells exomiRNAS in PCa tissue tumours according to tumor grade and, may be useful for PCa prognosis purposes. All these PCa tumours genes could be potential targets pointing to an alteration in the repressive machinery of exomiRNA-tRNA-mediated gene synthesis and proliferation.

### Supplementary Information

**Additional file 1.** Fold change of aberrantly expressed exomiRNAs resulted from comparations of: LNCaP vs RWPE-1; PC-3 vs RWPE-1, and (LNCaP and PC-3) vs RWPE-1.

**Additional file 2.** Full Reactome pathway enrichment analysis list of pathways affected by downregulated exomiRNAs in PCa cells and putative implicated target genes.

**Additional file 3.** Full Reactome pathway enrichment analysis list of pathways affected by downregulated exomiRNAs in PC-3 cells vs LNCaP cell lines and putative implicated target genes.

**Additional file 4.** Full Reactome pathway enrichment analysis list of pathways affected by upregulated exomiRNAs in PC-3 cells vs LNCaP cell lines and putative implicated target genes.

**Additional file 5: Figure S1. **miRNeT Schematic representation of the interaction network of exomiRNAs with their targets-genes. **a** Network of exomiRNAs target genes regulated by at least 21/36 downregulated miRNAs in PCa cells vs RWPE-1 cells. **b** Network of exomiRNAs target genes regulated by at least 8/14 exomiRNAs downregulated miRNAs in PC-3 vs LNCaP cell line. **c** Network of exomiRNAs target genes regulated by at least 29/53 exomiRNAs downregulated miRNAs in PC-3 vs LNCaP cell line. **Figure S2. **Spearman correlation matrix. The Correlation map was plotted using significance levels for Spearman´s test performed with deregulated exomiRNAs and selected target genes. **a** Diagnostic study. **b** Prognostic study. Positive correlations are displayed in grading-blue and negative correlations in grading-red colour. Correlations with p-value ≥0.05 are considered insignificant and are left blank. Colour intensity and the size of the circle are proportional to the correlation coefficients. On the right side of the correlogram, the legend colour shows the correlation coefficients. **Figure S3. a** Boxplot showing mRNAs expression of *CDK6*, *TNRC6B*, *AGO1*, *AGO3,* and *TNRC6A* in prostate tumour tissue samples of patients stratified according to the ISUP-GG classification retrieved from the TCGA-PRAD database. Box plots show the median, quartiles, and extreme values. Different lettering over the boxes indicates statistical differences. Significant differences are established at p < 0.05. **b **Boxplot showing mRNA expression of *CDK6*, *TNRC6B,* and *AGO1* in prostate tumour tissue samples of patients stratified according to affected lymph nodes split into N0 (no cancer in nearby lymph nodes) and N1 (cancer cells in 1 nearby lymph node) as stated in TCGA-PRAD database. Box plots show the median, quartiles, and extreme values. Different letter over the boxes indicates statistical differences. Significant differences are established at p < 0.05.

**Additional file 6: Table S1**. Patients’ characteristic used for diagnosis approach analysis. **Table S2. **Prediction of microRNA binding sites by miRWalk. **Table S3**. Patients’ characteristic used for prognosis approach analysis.

## Data Availability

The original contributions presented in the study are included in the Supplementary Material. Further inquiries can be directed to the corresponding author.

## Abbreviations

AGO1: Argonaute RISC Component 1
AGO3: Argonaute RISC Component 3
AGO4: Argonaute RISC Component 4
AR: Androgen receptor
AUC: Area under the curve
BPE: Bovine pituitary extract
CART: Classification and regression trees
CD9: Cluster of Differentiation 9
CD81: Cluster of Differentiation 81
CDH2: Cadherin-2
CDKN1A: Cyclin-dependent kinase inhibitor 1
CDKN2A: Cyclin-dependent kinase 4 inhibitor A
CDKN2B: Cyclin-dependent kinase 4 inhibitor B
CDK6: Cell division protein kinase 6
cDNA: Complementary Desoxyribonucleic acid
CI: Confidential Interval
DDX6: DEAD-Box Helicase 6
EMT: Epithelial-mesenchymal transition
exomiRNAs: Exovesicles-derived microRNAs
EVs: Extracellular vesicles
FBS: Fetal Bovine Serum
HSP70: Heat-shock protein 70
ISEV: International Society of Extracellular Vesicles
ISUP-GG: International Society of Urological Pathology-Gleason Grade
KMT2A: Histone-lysine N-methyltransferase 2A
KMT2B: Histone-lysine N-methyltransferase 2B
KMT2C: Histone-lysine N-methyltransferase 2C
KMT2D: Histone-lysine N-methyltransferase 2D
KMT2E: Histone-lysine N-methyltransferase 2E
LIMD1: LIM domain containing 1
MAPK: Mitogen-activated protein kinase
ml: Millilitres
mRNA: Messenger ribonucleic acid
NLK: Nemo Like Kinase
nm: Nanometres
N-PP: Non-pathologic prostate
PCa: Prostate cancer
PT: Prostate Tumour
PSA: Prostate Serum Antigen
PTEN: Phosphatase and tensin homolog
qRT-PCR: Quantitive Real Time-Polymerasa Chain Reaction
RB1: Retinoblastoma
rhEGF: Recombinant human epidermal growth factor
RISC: RNA-induced silencing complex
RNA: Ribonucleic acid
ROC: Receiver operating characteristic
RUNX1: Runt-related transcription factor 1
SETD1A: SET domain containing 1A
SETD1B: SET domain containing 1B
siRNA: Short-interfering ribonucleic acid
SNPs: Single nucleotide polymorphisms
TCF12: Transcription Factor 12
TCGA-PRAD: The Cancer Genome Atlas Prostate Adenocarcinoma
TEM: Transmission electron microscopy
TNRC6A: Trinucleotide repeat-containing gene 6A
TNRC6B: Trinucleotide repeat-containing gene 6B
UTRs: Untranslated regions
VIM: Vimentin
μm: Micrometres

## References

[1] Siegel RL, Miller KD, Fuchs HE, Jemal A (2021). Cancer statistics, 2021. CA Cancer J Clin.

[2] Klusa D, Lohaus F, Furesi G, Rauner M, Benešová M, Krause M (2021). Metastatic spread in prostate cancer patients influencing radiotherapy response. Front Oncol.

[3] Saranyutanon S, Deshmukh SK, Dasgupta S, Pai S, Singh S, Singh AP (2020). Cellular and molecular progression of prostate cancer: models for basic and preclinical research. Cancers.

[4] Théry C, Witwer KW, Aikawa E, Alcaraz MJ, Anderson JD, Andriantsitohaina R (2018). Minimal information for studies of extracellular vesicles 2018 (MISEV2018): a position statement of the international society for extracellular vesicles and update of the MISEV2014 guidelines. J Extracell Vesicles.

[5] Maas SLN, Breakefield XO, Weaver AM (2017). Extracellular vesicles: unique intercellular delivery vehicles. Trends Cell Biol.

[6] Tkach M, Théry C (2016). Communication by extracellular vesicles: where we are and where we need to go. Cell.

[7] Ramirez-Garrastacho M, Bajo-Santos C, Line A, Martens-Uzunova ES, de la Fuente JM, Moros M (2021). Extracellular vesicles as a source of prostate cancer biomarkers in liquid biopsies: a decade of research. Br J Cancer.

[8] Hessvik NP, Phuyal S, Brech A, Sandvig K, Llorente A (2012). Profiling of microRNAs in exosomes released from PC-3 prostate cancer cells. Biochim Biophys Acta Gene Regul Mech.

[9] Santo GD, Frasca M, Bertoli G, Castiglioni I, Cava C (2022). Identification of key miRNAs in prostate cancer progression based on miRNA-mRNA network construction. Comput Struct Biotechnol J.

[10] Bhagirath D, Yang TL, Bucay N, Sekhon K, Majid S, Shahryari V (2018). microRNA-1246 is an exosomal biomarker for aggressive prostate cancer. Cancer Res.

[11] Ruiz-Plazas X, Altuna-Coy A, Alves-Santiago M, Vila-Barja J, Francesc García-Fontgivell J, Martínez-González S (2021). Liquid biopsy-based exo-oncomiRNAs can predict prostate cancer aggressiveness liquid biopsy-based. Cancer.

[12] Sticht C, De La Torre C, Parveen A, Gretz N (2018). Mirwalk: an online resource for prediction of microrna binding sites. PLoS ONE.

[13] Thiery G, Bernier J, Bergeron M (1995). A simple technique for staining of cell membranes with imidazole and osmium tetroxide. J Histochem Cytochem.

[14] Epstein JI, Egevad L, Amin MB, Delahunt B, Srigley JR, Humphrey PA (2016). The 2014 International Society of Urological Pathology (ISUP) consensus conference on Gleason grading of prostatic carcinoma: definition of grading patterns and proposal for a new grading system. Am J Surg Pathol.

[15] Weinmann L, Höck J, Ivacevic T, Ohrt T, Mütze J, Schwille P (2009). Importin 8 is a gene silencing factor that targets argonaute proteins to distinct mRNAs. Cell.

[16] Meister G, Landthaler M, Peters L, Chen PY, Urlaub H, Lührmann R (2005). Identification of novel argonaute-associated proteins. Curr Biol.

[17] Räsch F, Weber R, Izaurralde E, Igreja C (2020). 4E-T-bound mRNAs are stored in a silenced and deadenylated form. Genes Dev.

[18] Zarringhalam K, Tay Y, Kulkarni P, Bester AC, Pandolfi PP, Kulkarni RV (2017). Identification of competing endogenous RNAs of the tumor suppressor gene PTEN: a probabilistic approach. Sci Rep.

[19] Jamaspishvili T, Berman DM, Ross AE, Scher HI, De Marzo AM, Squire JA (2018). Clinical implications of PTEN loss in prostate cancer. Nat Rev Urol Nat Publ Group.

[20] Zhou X, Yang X, Sun X, Xu X, Li X, Guo Y (2019). Effect of PTEN loss on metabolic reprogramming in prostate cancer cells. Oncol Lett.

[21] Sun J, Zheng SL, Wiklund F, Isaacs SD, Li G, Wiley KE (2009). Sequence variants at 22q13 are associated with prostate cancer risk. Cancer Res.

[22] Tao S, Wang Z, Feng J, Hsu FC, Jin G, Kim ST (2012). A genome-wide search for loci interacting with known prostate cancer risk-associated genetic variants. Carcinogenesis.

[23] Merriel SWD, Pocock L, Gilbert E, Creavin S, Walter FM, Spencer A (2022). Systematic review and meta-analysis of the diagnostic accuracy of prostate-specific antigen (PSA) for the detection of prostate cancer in symptomatic patients. BMC Med.

[24] Goel S, Bergholz JS, Zhao JJ (2022). Targeting CDK4 and CDK6 in cancer. Nat Rev Cancer Nat Res.

[25] Wang G, Zheng L, Yu Z, Liao G, Lu L, Xu R (2012). Increased cyclin-dependent kinase 6 expression in bladder cancer. Oncol Lett.

[26] Lee KH, Lotterman C, Karikari C, Omura N, Feldmann G, Habbe N (2009). Epigenetic silencing of microRNA miR-107 regulates cyclin-dependent kinase 6 expression in pancreatic cancer. Pancreatology.

[27] Kollmann K, Briand C, Bellutti F, Schicher N, Blunder S, Zojer M (2019). The interplay of CDK4 and CDK6 in melanoma. Oncotarget.

[28] Lim JTE, Mansukhani M, Weinstein IB (2005). Cyclin-dependent kinase 6 associates with the androgen receptor and enhances its transcriptional activity in prostate cancer cells. Proc Natl Acad Sci.

[29] Wu C, Peng S, Pilie PG, Geng C, Park S, Manyam GC (2021). PARP and CDK4/6 inhibitor combination therapy induces apoptosis and suppresses neuroendocrine differentiation in prostate cancer. Mol Cancer Ther.

[30] Müller M, Fazi F, Ciaudo C (2020). Argonaute proteins: from structure to function in development and pathological cell fate determination. Front Cell Dev Biol.

[31] Huang V, Zheng J, Qi Z, Wang J, Place RF, Yu J (2013). Ago1 interacts with RNA polymerase II and binds to the promoters of actively transcribed genes in human cancer cells. PLoS Genet.

[32] Henderson RJ, Eastham JA, Culkin DJ, Kattan MW, Whatley T, Mata J (1997). Prostate-specific antigen (PSA) and PSA density: racial differences in men without prostate cancer. J Natl Cancer Inst.

