# Supplementary material for: In silico analysis of prognostic and diagnostic significance of target genes from prostate cancer cell lines derived exomicroRNAs
Source: Cancer Cell Int. 2023 Nov 17;23:275. doi: 10.1186/s12935-023-03123-1 (PMC10655318; doi:10.1186/s12935-023-03123-1)
Supplement: Supplementary file 2 — Additional file 2. Full Reactome pathway enrichment analysis list of pathways affected by downregulated exomiRNAs in PCa cells and putative implicated target genes. [file 12935_2023_3123_MOESM2_ESM.docx]

**Additional File 2**

| **Pathway** | **Hits/Total Genes Involved** | **Adjusted *p*-value** | **Genes** |
| --- | --- | --- | --- |
| **ESR-mediated Signaling (R-HSA-8939211)** | **7/188** | **< 0,001** | **YY1,NCOA3,AGO1,TNRC6A,IGF1R,UHMK1,TNRC6B** |
| **RUNX1 Regulates Genes Involved In Megakaryocyte Differentiation And Platelet Function (R-HSA-8936459)** | **5/66** | **< 0,001** | **KMT2A,AGO1,THBS1,TNRC6A,TNRC6B** |
| **Post-transcriptional Silencing By Small RNAs (R-HSA-426496)** | **3/7** | **< 0,001** | **AGO1,TNRC6A,TNRC6B** |
| **Transcriptional Regulation By TP53 (R-HSA-3700989)** | **8/354** | **< 0,001** | **BTG2,CNOT1,AGO1,MDM2,PMAIP1,HIPK1,TNRC6A,TNRC6B** |
| **Oncogene Induced Senescence (R-HSA-2559585)** | **4/33** | **< 0,001** | **AGO1,MDM2,TNRC6A,TNRC6B** |
| **Signaling By Nuclear Receptors (R-HSA-9006931)** | **7/260** | **< 0,001** | **YY1,NCOA3,AGO1,TNRC6A,IGF1R,UHMK1,TNRC6B** |
| **Competing Endogenous RNAs (ceRNAs) Regulate PTEN Translation (R-HSA-8948700)** | **3/10** | **< 0,001** | **AGO1,TNRC6A,TNRC6B** |
| **Regulation Of PTEN mRNA Translation (R-HSA-8943723)** | **3/11** | **< 0,001** | **AGO1,TNRC6A,TNRC6B** |
| **Generic Transcription Pathway (R-HSA-212436)** | **12/1190** | **< 0,001** | **YY1,BTG2,BCL2L11,KMT2A,CNOT1,AGO1,MDM2,PMAIP1,HIPK1,THBS1,TNRC6A,TNRC6B** |
| **Gene Expression (Transcription) (R-HSA-74160)** | **13/1449** | **< 0,001** | **BTG2,KMT2A,DICER1,HIPK1,THBS1,YY1,BCL2L11,CNOT1,AGO1,MDM2,PMAIP1,TNRC6A,TNRC6B** |
| **Estrogen-dependent Gene Expression (R-HSA-9018519)** | **5/119** | **< 0,001** | **YY1,NCOA3,AGO1,TNRC6A,TNRC6B** |
| **Regulation Of RUNX1 Expression And Activity (R-HSA-8934593)** | **3/17** | **< 0,001** | **AGO1,TNRC6A,TNRC6B** |
| **RNA Polymerase II Transcription (R-HSA-73857)** | **12/1312** | **< 0,001** | **YY1,BTG2,BCL2L11,KMT2A,CNOT1,AGO1,MDM2,PMAIP1,HIPK1,THBS1,TNRC6A,TNRC6B** |
| **Beta-catenin Independent WNT Signaling (R-HSA-3858494)** | **5/142** | **< 0,001** | **NCOA3,AGO1,CLTC,TNRC6A,TNRC6B** |
| **MAPK6/MAPK4 Signaling (R-HSA-5687128)** | **4/90** | **< 0,001** | **NCOA3,AGO1,TNRC6A,TNRC6B** |
| **Regulation Of MECP2 Expression And Activity (R-HSA-9022692)** | **3/31** | **< 0,001** | **AGO1,TNRC6A,TNRC6B** |
| **Oxidative Stress Induced Senescence (R-HSA-2559580)** | **4/93** | **< 0,001** | **AGO1,MDM2,TNRC6A,TNRC6B** |
| **NR1H3 And NR1H2 Regulate Gene Expression Linked To Cholesterol Transport And Efflux (R-HSA-9029569)** | **3/36** | **< 0,001** | **AGO1,TNRC6A,TNRC6B** |
| **Transcriptional Regulation By RUNX1 (R-HSA-8878171)** | **5/204** | **< 0,001** | **KMT2A,AGO1,THBS1,TNRC6A,TNRC6B** |
| **Transcriptional Regulation By VENTX (R-HSA-8853884)** | **3/39** | **< 0,001** | **AGO1,TNRC6A,TNRC6B** |
| **Gene Silencing By RNA (R-HSA-211000)** | **4/108** | **< 0,001** | **AGO1,DICER1,TNRC6A,TNRC6B** |
| **NR1H2 And NR1H3-mediated Signaling (R-HSA-9024446)** | **3/46** | **0,001** | **AGO1,TNRC6A,TNRC6B** |
| **Small Interfering RNA (siRNA) Biogenesis (R-HSA-426486)** | **2/9** | **0,001** | **AGO1,DICER1** |
| **BH3-only Proteins Associate With And Inactivate Anti-Apoptotic BCL-2 Members (R-HSA-111453)** | **2/9** | **0,001** | **BCL2L11,PMAIP1** |
| **Transcriptional Regulation By MECP2 (R-HSA-8986944)** | **3/60** | **0,002** | **AGO1,TNRC6A,TNRC6B** |
| **Ca2+ Pathway (R-HSA-4086398)** | **3/61** | **0,002** | **AGO1,TNRC6A,TNRC6B** |
| **Pre-NOTCH Transcription And Translation (R-HSA-1912408)** | **3/62** | **0,002** | **AGO1,TNRC6A,TNRC6B** |
| **Signaling By WNT (R-HSA-195721)** | **5/294** | **0,003** | **NCOA3,AGO1,CLTC,TNRC6A,TNRC6B** |
| **Cellular Senescence (R-HSA-2559583)** | **4/165** | **0,003** | **AGO1,MDM2,TNRC6A,TNRC6B** |
| **Pre-NOTCH Expression And Processing (R-HSA-1912422)** | **3/78** | **0,004** | **AGO1,TNRC6A,TNRC6B** |
| **TP53 Regulates Metabolic Genes (R-HSA-5628897)** | **3/81** | **0,004** | **AGO1,TNRC6A,TNRC6B** |
| **LDL Clearance (R-HSA-8964038)** | **2/18** | **0,004** | **CLTC,LDLR** |
| **TP53 Regulates Transcription Of Additional Cell Cycle Genes With Uncertain Roles In P53 Pathway (R-HSA-6804115)** | **2/21** | **0,006** | **BTG2,CNOT1** |
| **MicroRNA (miRNA) Biogenesis (R-HSA-203927)** | **2/24** | **0,007** | **AGO1,DICER1** |
| **Activation Of BH3-only Proteins (R-HSA-114452)** | **2/30** | **0,011** | **BCL2L11,PMAIP1** |
| **PIP3 Activates AKT Signaling (R-HSA-1257604)** | **4/268** | **0,014** | **AGO1,MDM2,TNRC6A,TNRC6B** |
| **Plasma Lipoprotein Clearance (R-HSA-8964043)** | **2/35** | **0,015** | **CLTC,LDLR** |
| **PTEN Regulation (R-HSA-6807070)** | **3/139** | **0,017** | **AGO1,TNRC6A,TNRC6B** |
| **Signal Transduction (R-HSA-162582)** | **12/2465** | **0,018** | **YY1,BCL2L11,NCOA3,TAOK1,AGO1,CLTC,MDM2,THBS1,TNRC6A,IGF1R,UHMK1,TNRC6B** |
| **Intracellular Signaling By Second Messengers (R-HSA-9006925)** | **4/306** | **0,020** | **AGO1,MDM2,TNRC6A,TNRC6B** |
| **MAPK Family Signaling Cascades (R-HSA-5683057)** | **4/318** | **0,022** | **NCOA3,AGO1,TNRC6A,TNRC6B** |
| **TP53 Regulates Transcription Of Cell Cycle Genes (R-HSA-6791312)** | **2/49** | **0,025** | **BTG2,CNOT1** |
| **Intrinsic Pathway For Apoptosis (R-HSA-109606)** | **2/55** | **0,030** | **BCL2L11,PMAIP1** |
| **Signaling By NOTCH (R-HSA-157118)** | **3/203** | **0,043** | **AGO1,TNRC6A,TNRC6B** |
| **Plasma Lipoprotein Assembly, Remodeling, And Clearance (R-HSA-174824)** | **2/71** | **0,048** | **CLTC,LDLR** |
| **Extra-nuclear Estrogen Signaling (R-HSA-9009391)** | **2/73** | **0,048** | **IGF1R,UHMK1** |
| **Activation Of NOXA And Translocation To Mitochondria (R-HSA-111448)** | **1/5** | **0,048** | **PMAIP1** |
| **RUNX3 Regulates BCL2L11 (BIM) Transcription (R-HSA-8952158)** | **1/5** | **0,048** | **BCL2L11** |
| **Chylomicron Clearance (R-HSA-8964026)** | **1/5** | **0,048** | **LDLR** |
| **Transcriptional Regulation By Small RNAs (R-HSA-5578749)** | **2/76** | **0,049** | **AGO1,TNRC6A** |
| Entry Of Influenza Virion Into Host Cell Via Endocytosis (R-HSA-168275) | 1/6 | 0,055 | CLTC |
| PCP/CE Pathway (R-HSA-4086400) | 2/88 | 0,062 | NCOA3,CLTC |
| Regulation Of TP53 Activity Thru Phosphorylation (R-HSA-6804756) | 2/90 | 0,064 | MDM2,HIPK1 |
| Activation Of HOX Genes During Differentiation (R-HSA-5619507) | 2/91 | 0,064 | YY1,NCOA3 |
| Transcriptional Regulation By RUNX3 (R-HSA-8878159) | 2/95 | 0,066 | BCL2L11,MDM2 |
| Coenzyme A Biosynthesis (R-HSA-196783) | 1/8 | 0,066 | PANK3 |
| SHC-related Events Triggered By IGF1R (R-HSA-2428933) | 1/8 | 0,066 | IGF1R |
| Formation Of Annular Gap Junctions (R-HSA-196025) | 1/9 | 0,073 | CLTC |
| Cargo Recognition For Clathrin-Mediated Endocytosis (R-HSA-8856825) | 2/104 | 0,075 | CLTC,LDLR |
| Gap Junction Degradation (R-HSA-190873) | 1/10 | 0,078 | CLTC |
| WNT5A-dependent Internalization Of FZD2, FZD5 And ROR2 (R-HSA-5140745) | 1/11 | 0,084 | CLTC |
| ALK Mutants Bind TKIs (R-HSA-9700645) | 1/12 | 0,090 | CLTC |
| TFAP2 (AP-2) Family Regulates Transcription Of Growth Factors And Their Receptors (R-HSA-8866910) | 1/13 | 0,096 | YY1 |
| AKT Phosphorylates Targets In Cytosol (R-HSA-198323) | 1/14 | 0,099 | MDM2 |
| Physiological Factors (R-HSA-5578768) | 1/14 | 0,099 | HIPK1 |
| Retrograde Neurotrophin Signaling (R-HSA-177504) | 1/14 | 0,099 | CLTC |
| WNT5A-dependent Internalization Of FZD4 (R-HSA-5099900) | 1/15 | 0,103 | CLTC |
| YAP1- And WWTR1 (TAZ)-stimulated Gene Expression (R-HSA-2032785) | 1/15 | 0,103 | HIPK1 |
| FOXO-mediated Transcription Of Cell Death Genes (R-HSA-9614657) | 1/16 | 0,106 | BCL2L11 |
| VLDLR Internalisation And Degradation (R-HSA-8866427) | 1/16 | 0,106 | CLTC |
| Clathrin-mediated Endocytosis (R-HSA-8856828) | 2/142 | 0,110 | CLTC,LDLR |
| Vitamin B5 (Pantothenate) Metabolism (R-HSA-199220) | 1/17 | 0,110 | PANK3 |
| Regulation Of TP53 Activity Thru Methylation (R-HSA-6804760) | 1/19 | 0,117 | MDM2 |
| SUMOylation Of Transcription Factors (R-HSA-3232118) | 1/19 | 0,117 | MDM2 |
| Nuclear Events Stimulated By ALK Signaling In Cancer (R-HSA-9725371) | 1/19 | 0,117 | CLTC |
| Syndecan Interactions (R-HSA-3000170) | 1/20 | 0,120 | THBS1 |
| TP53 Regulates Transcription Of Genes Involved In Cytochrome C Release (R-HSA-6803204) | 1/20 | 0,120 | PMAIP1 |
| Regulation Of TP53 Activity (R-HSA-5633007) | 2/157 | 0,121 | MDM2,HIPK1 |
| Citric Acid Cycle (TCA Cycle) (R-HSA-71403) | 1/21 | 0,121 | LDLR |
| Deregulated CDK5 Triggers Neurodegenerative Pathways In Alzheimers Disease Models (R-HSA-8862803) | 1/21 | 0,121 | BCL2L11 |
| Glutamate Neurotransmitter Release Cycle (R-HSA-210500) | 1/23 | 0,131 | SLC38A2 |
| Defective Intrinsic Pathway For Apoptosis (R-HSA-9734009) | 1/24 | 0,133 | BCL2L11 |
| Estrogen-dependent Nuclear Events Downstream Of ESR-membrane Signaling (R-HSA-9634638) | 1/24 | 0,133 | UHMK1 |
| Gap Junction Trafficking (R-HSA-190828) | 1/25 | 0,134 | CLTC |
| Interleukin-7 Signaling (R-HSA-1266695) | 1/25 | 0,134 | BRWD1 |
| Deadenylation Of mRNA (R-HSA-429947) | 1/25 | 0,134 | CNOT1 |
| Apoptosis (R-HSA-109581) | 2/178 | 0,136 | BCL2L11,PMAIP1 |
| Constitutive Signaling By AKT1 E17K In Cancer (R-HSA-5674400) | 1/26 | 0,136 | MDM2 |
| Gap Junction Trafficking And Regulation (R-HSA-157858) | 1/27 | 0,138 | CLTC |
| Transport Of Small Molecules (R-HSA-382551) | 4/706 | 0,138 | WNK1,CLTC,LDLR,SLC38A2 |
| Metabolism Of Vitamins And Cofactors (R-HSA-196854) | 2/186 | 0,138 | PANK3,LDLR |
| Diseases Of Signal Transduction By Growth Factor Receptors And Second Messengers (R-HSA-5663202) | 3/424 | 0,138 | BCL2L11,CLTC,MDM2 |
| Recycling Pathway Of L1 (R-HSA-437239) | 1/28 | 0,138 | CLTC |
| Cellular Responses To Stress (R-HSA-2262752) | 4/722 | 0,145 | AGO1,MDM2,TNRC6A,TNRC6B |
| Synthesis Of Active Ubiquitin: Roles Of E1 And E2 Enzymes (R-HSA-8866652) | 1/30 | 0,145 | UBE2W |
| Glutamate Binding, Activation Of AMPA Receptors And Synaptic Plasticity (R-HSA-399721) | 1/31 | 0,148 | MDM2 |
| Cellular Responses To Stimuli (R-HSA-8953897) | 4/736 | 0,149 | AGO1,MDM2,TNRC6A,TNRC6B |
| Amino Acid Transport Across Plasma Membrane (R-HSA-352230) | 1/33 | 0,154 | SLC38A2 |
| Lysosome Vesicle Biogenesis (R-HSA-432720) | 1/34 | 0,156 | CLTC |
| Programmed Cell Death (R-HSA-5357801) | 2/208 | 0,156 | BCL2L11,PMAIP1 |
| Transcriptional Regulation By AP-2 (TFAP2) Family Of Transcription Factors (R-HSA-8864260) | 1/36 | 0,157 | YY1 |
| Regulation Of TP53 Degradation (R-HSA-6804757) | 1/36 | 0,157 | MDM2 |
| FLT3 Signaling (R-HSA-9607240) | 1/37 | 0,157 | BCL2L11 |
| RHOV GTPase Cycle (R-HSA-9013424) | 1/37 | 0,157 | CLTC |
| Regulation Of TP53 Expression And Degradation (R-HSA-6806003) | 1/37 | 0,157 | MDM2 |
| DNA Damage Recognition In GG-NER (R-HSA-5696394) | 1/37 | 0,157 | YY1 |
| Defective B3GALTL Causes PpS (R-HSA-5083635) | 1/37 | 0,157 | THBS1 |
| O-glycosylation Of TSR Domain-Containing Proteins (R-HSA-5173214) | 1/38 | 0,160 | THBS1 |
| RHOU GTPase Cycle (R-HSA-9013420) | 1/39 | 0,161 | CLTC |
| SUMOylation Of Ubiquitinylation Proteins (R-HSA-3232142) | 1/39 | 0,161 | MDM2 |
| Non-integrin membrane-ECM Interactions (R-HSA-3000171) | 1/41 | 0,166 | THBS1 |
| Signaling By Receptor Tyrosine Kinases (R-HSA-9006934) | 3/496 | 0,166 | CLTC,THBS1,IGF1R |
| Retinoid Metabolism And Transport (R-HSA-975634) | 1/43 | 0,172 | LDLR |
| Chromatin Modifying Enzymes (R-HSA-3247509) | 2/238 | 0,172 | KMT2A,BRWD1 |
| TP53 Regulates Transcription Of Cell Death Genes (R-HSA-5633008) | 1/44 | 0,173 | PMAIP1 |
| Transmission Across Chemical Synapses (R-HSA-112315) | 2/246 | 0,179 | MDM2,SLC38A2 |
| PKMTs Methylate Histone Lysines (R-HSA-3214841) | 1/47 | 0,179 | KMT2A |
| Metabolism Of Fat-Soluble Vitamins (R-HSA-6806667) | 1/47 | 0,179 | LDLR |
| EPH-ephrin Mediated Repulsion Of Cells (R-HSA-3928665) | 1/49 | 0,185 | CLTC |
| Neurotransmitter Release Cycle (R-HSA-112310) | 1/50 | 0,187 | SLC38A2 |
| IRS-related Events Triggered By IGF1R (R-HSA-2428928) | 1/51 | 0,189 | IGF1R |
| IGF1R Signaling Cascade (R-HSA-2428924) | 1/52 | 0,189 | IGF1R |
| Signaling By PDGF (R-HSA-186797) | 1/52 | 0,189 | THBS1 |
| Signaling By Type 1 Insulin-like Growth Factor 1 Receptor (IGF1R) (R-HSA-2404192) | 1/53 | 0,189 | IGF1R |
| Pyruvate Metabolism And Citric Acid (TCA) Cycle (R-HSA-71406) | 1/54 | 0,189 | LDLR |
| Regulation Of RUNX3 Expression And Activity (R-HSA-8941858) | 1/54 | 0,189 | MDM2 |
| Cell Cycle Checkpoints (R-HSA-69620) | 2/271 | 0,189 | TAOK1,MDM2 |
| Golgi Associated Vesicle Biogenesis (R-HSA-432722) | 1/55 | 0,189 | CLTC |
| Regulation Of Cholesterol Biosynthesis By SREBP (SREBF) (R-HSA-1655829) | 1/55 | 0,189 | INSIG1 |
| Signaling By ALK Fusions And Activated Point Mutants (R-HSA-9725370) | 1/55 | 0,189 | CLTC |
| Stabilization Of P53 (R-HSA-69541) | 1/56 | 0,189 | MDM2 |
| Deadenylation-dependent mRNA Decay (R-HSA-429914) | 1/56 | 0,189 | CNOT1 |
| NRAGE Signals Death Thru JNK (R-HSA-193648) | 1/57 | 0,191 | BCL2L11 |
| Deubiquitination (R-HSA-5688426) | 2/279 | 0,192 | YY1,MDM2 |
| Transcriptional Regulation Of Granulopoiesis (R-HSA-9616222) | 1/59 | 0,195 | KMT2A |
| Signaling By BRAF And RAF1 Fusions (R-HSA-6802952) | 1/62 | 0,202 | BCL2L11 |
| FOXO-mediated Transcription (R-HSA-9614085) | 1/65 | 0,208 | BCL2L11 |
| p53-Dependent G1 DNA Damage Response (R-HSA-69563) | 1/65 | 0,208 | MDM2 |
| Integrin Cell Surface Interactions (R-HSA-216083) | 1/66 | 0,210 | THBS1 |
| G1/S DNA Damage Checkpoints (R-HSA-69615) | 1/67 | 0,211 | MDM2 |
| Diseases Associated With O-glycosylation Of Proteins (R-HSA-3906995) | 1/69 | 0,216 | THBS1 |
| trans-Golgi Network Vesicle Budding (R-HSA-199992) | 1/71 | 0,218 | CLTC |
| Diseases Of Programmed Cell Death (R-HSA-9645723) | 1/71 | 0,218 | BCL2L11 |
| Cell Death Signaling Via NRAGE, NRIF And NADE (R-HSA-204998) | 1/74 | 0,225 | BCL2L11 |
| Protein Ubiquitination (R-HSA-8852135) | 1/76 | 0,228 | UBE2W |
| Signaling By Rho GTPases (R-HSA-194315) | 3/644 | 0,228 | NCOA3,TAOK1,CLTC |
| Oncogenic MAPK Signaling (R-HSA-6802957) | 1/78 | 0,232 | BCL2L11 |
| Signaling By Rho GTPases, Miro GTPases And RHOBTB3 (R-HSA-9716542) | 3/660 | 0,237 | NCOA3,TAOK1,CLTC |
| Global Genome Nucleotide Excision Repair (GG-NER) (R-HSA-5696399) | 1/82 | 0,240 | YY1 |
| Transcriptional Regulation Of White Adipocyte Differentiation (R-HSA-381340) | 1/84 | 0,243 | NCOA3 |
| EPH-Ephrin Signaling (R-HSA-2682334) | 1/91 | 0,260 | CLTC |
| Cytokine Signaling In Immune System (R-HSA-1280215) | 3/702 | 0,260 | BCL2L11,BRWD1,MCL1 |
| Unattached Kinetochores Signal Amplification Via A MAD2 Inhibitory Signal (R-HSA-141444) | 1/93 | 0,260 | TAOK1 |
| RAC3 GTPase Cycle (R-HSA-9013423) | 1/93 | 0,260 | NCOA3 |
| Developmental Biology (R-HSA-1266738) | 4/1073 | 0,260 | YY1,KMT2A,NCOA3,CLTC |
| UCH Proteinases (R-HSA-5689603) | 1/95 | 0,260 | YY1 |
| P75 NTR Receptor-Mediated Signaling (R-HSA-193704) | 1/95 | 0,260 | BCL2L11 |
| EML4 And NUDC In Mitotic Spindle Formation (R-HSA-9648025) | 1/97 | 0,262 | TAOK1 |
| Visual Phototransduction (R-HSA-2187338) | 1/98 | 0,262 | LDLR |
| RUNX1 Regulates Transcription Of Genes Involved In Differentiation Of HSCs (R-HSA-8939236) | 1/98 | 0,262 | KMT2A |
| L1CAM Interactions (R-HSA-373760) | 1/99 | 0,263 | CLTC |
| Stimuli-sensing Channels (R-HSA-2672351) | 1/100 | 0,264 | WNK1 |
| Neuronal System (R-HSA-112316) | 2/386 | 0,264 | MDM2,SLC38A2 |
| Transport Of Inorganic Cations/Anions And Amino Acids/Oligopeptides (R-HSA-425393) | 1/104 | 0,269 | SLC38A2 |
| MHC Class II Antigen Presentation (R-HSA-2132295) | 1/104 | 0,269 | CLTC |
| PI3K/AKT Signaling In Cancer (R-HSA-2219528) | 1/105 | 0,269 | MDM2 |
| Resolution Of Sister Chromatid Cohesion (R-HSA-2500257) | 1/106 | 0,269 | TAOK1 |
| O-linked Glycosylation (R-HSA-5173105) | 1/107 | 0,269 | THBS1 |
| Interleukin-4 And Interleukin-13 Signaling (R-HSA-6785807) | 1/107 | 0,269 | MCL1 |
| Nucleotide Excision Repair (R-HSA-5696398) | 1/108 | 0,270 | YY1 |
| Mitotic Spindle Checkpoint (R-HSA-69618) | 1/110 | 0,272 | TAOK1 |
| Signaling By NTRK1 (TRKA) (R-HSA-187037) | 1/114 | 0,280 | CLTC |
| PPARA Activates Gene Expression (R-HSA-1989781) | 1/116 | 0,282 | NCOA3 |
| Regulation Of Lipid Metabolism By PPARalpha (R-HSA-400206) | 1/118 | 0,285 | NCOA3 |
| RHO GTPases Activate Formins (R-HSA-5663220) | 1/119 | 0,286 | TAOK1 |
| SARS-CoV-2 Activates/Modulates Innate And Adaptive Immune Responses (R-HSA-9705671) | 1/121 | 0,288 | G3BP1 |
| Metabolism Of Water-Soluble Vitamins And Cofactors (R-HSA-196849) | 1/122 | 0,289 | PANK3 |
| Platelet Degranulation (R-HSA-114608) | 1/125 | 0,292 | THBS1 |
| Cardiac Conduction (R-HSA-5576891) | 1/126 | 0,292 | HIPK1 |
| RHO GTPase Cycle (R-HSA-9012999) | 2/441 | 0,292 | NCOA3,CLTC |
| Response To Elevated Platelet Cytosolic Ca2+ (R-HSA-76005) | 1/130 | 0,298 | THBS1 |
| Signaling By Interleukins (R-HSA-449147) | 2/453 | 0,299 | BRWD1,MCL1 |
| Signaling By NTRKs (R-HSA-166520) | 1/132 | 0,299 | CLTC |
| Death Receptor Signaling (R-HSA-73887) | 1/139 | 0,311 | BCL2L11 |
| Diseases Of Glycosylation (R-HSA-3781865) | 1/143 | 0,317 | THBS1 |
| Metabolism Of Steroids (R-HSA-8957322) | 1/153 | 0,334 | INSIG1 |
| Influenza Infection (R-HSA-168255) | 1/157 | 0,340 | CLTC |
| Disease (R-HSA-1643685) | 5/1736 | 0,344 | BCL2L11,CLTC,G3BP1,MDM2,THBS1 |
| Citric Acid (TCA) Cycle And Respiratory Electron Transport (R-HSA-1428517) | 1/163 | 0,347 | LDLR |
| SUMO E3 Ligases SUMOylate Target Proteins (R-HSA-3108232) | 1/168 | 0,354 | MDM2 |
| Separation Of Sister Chromatids (R-HSA-2467813) | 1/170 | 0,356 | TAOK1 |
| SUMOylation (R-HSA-2990846) | 1/174 | 0,361 | MDM2 |
| Ion Channel Transport (R-HSA-983712) | 1/175 | 0,361 | WNK1 |
| mRNA Splicing - Major Pathway (R-HSA-72163) | 1/181 | 0,369 | HNRNPU |
| Neurotransmitter Receptors And Postsynaptic Signal Transmission (R-HSA-112314) | 1/182 | 0,369 | MDM2 |
| Post-translational Protein Modification (R-HSA-597592) | 4/1383 | 0,369 | YY1,UBE2W,MDM2,THBS1 |
| Mitotic Prometaphase (R-HSA-68877) | 1/186 | 0,372 | TAOK1 |
| mRNA Splicing (R-HSA-72172) | 1/189 | 0,375 | HNRNPU |
| SARS-CoV-2-host Interactions (R-HSA-9705683) | 1/196 | 0,382 | G3BP1 |
| Muscle Contraction (R-HSA-397014) | 1/196 | 0,382 | HIPK1 |
| Ub-specific Processing Proteases (R-HSA-5689880) | 1/201 | 0,388 | MDM2 |
| Membrane Trafficking (R-HSA-199991) | 2/599 | 0,397 | CLTC,LDLR |
| Immune System (R-HSA-168256) | 5/1943 | 0,416 | UBE2W,BCL2L11,CLTC,BRWD1,MCL1 |
| Vesicle-mediated Transport (R-HSA-5653656) | 2/637 | 0,425 | CLTC,LDLR |
| Mitotic Anaphase (R-HSA-68882) | 1/232 | 0,426 | TAOK1 |
| Mitotic Metaphase And Anaphase (R-HSA-2555396) | 1/233 | 0,426 | TAOK1 |
| Cell Cycle (R-HSA-1640170) | 2/654 | 0,433 | TAOK1,MDM2 |
| Processing Of Capped Intron-Containing Pre-mRNA (R-HSA-72203) | 1/242 | 0,435 | HNRNPU |
| Metabolism Of RNA (R-HSA-8953854) | 2/666 | 0,435 | CNOT1,HNRNPU |
| SLC-mediated Transmembrane Transport (R-HSA-425407) | 1/247 | 0,435 | SLC38A2 |
| Diseases Of Metabolism (R-HSA-5668914) | 1/247 | 0,435 | THBS1 |
| Platelet Activation, Signaling And Aggregation (R-HSA-76002) | 1/254 | 0,443 | THBS1 |
| RHO GTPase Effectors (R-HSA-195258) | 1/269 | 0,460 | TAOK1 |
| SARS-CoV-2 Infection (R-HSA-9694516) | 1/283 | 0,475 | G3BP1 |
| Metabolism Of Lipids (R-HSA-556833) | 2/732 | 0,475 | INSIG1,NCOA3 |
| Adaptive Immune System (R-HSA-1280218) | 2/733 | 0,475 | UBE2W,CLTC |
| Extracellular Matrix Organization (R-HSA-1474244) | 1/291 | 0,479 | THBS1 |
| Antigen Processing: Ubiquitination And Proteasome Degradation (R-HSA-983168) | 1/307 | 0,496 | UBE2W |
| DNA Repair (R-HSA-73894) | 1/310 | 0,497 | YY1 |
| SARS-CoV Infections (R-HSA-9679506) | 1/369 | 0,559 | G3BP1 |
| Class I MHC Mediated Antigen Processing And Presentation (R-HSA-983169) | 1/378 | 0,565 | UBE2W |
| M Phase (R-HSA-68886) | 1/380 | 0,565 | TAOK1 |
| Metabolism Of Proteins (R-HSA-392499) | 4/1890 | 0,568 | YY1,UBE2W,MDM2,THBS1 |
| Infectious Disease (R-HSA-5663205) | 2/961 | 0,609 | CLTC,G3BP1 |
| Metabolism (R-HSA-1430728) | 4/2049 | 0,631 | PANK3,INSIG1,NCOA3,LDLR |
| Axon Guidance (R-HSA-422475) | 1/519 | 0,672 | CLTC |
| Cell Cycle, Mitotic (R-HSA-69278) | 1/523 | 0,672 | TAOK1 |
| Nervous System Development (R-HSA-9675108) | 1/545 | 0,684 | CLTC |
| Hemostasis (R-HSA-109582) | 1/576 | 0,702 | THBS1 |
| Sensory Perception (R-HSA-9709957) | 1/616 | 0,723 | LDLR |
