# Supplementary material for: In silico analysis of prognostic and diagnostic significance of target genes from prostate cancer cell lines derived exomicroRNAs
Source: Cancer Cell Int. 2023 Nov 17;23:275. doi: 10.1186/s12935-023-03123-1 (PMC10655318; doi:10.1186/s12935-023-03123-1)
Supplement: Supplementary file 3 — Additional file 3. Full Reactome pathway enrichment analysis list of pathways affected by downregulated exomiRNAs in PC-3 cells vs LNCaP cell lines and putative implicated target genes. [file 12935_2023_3123_MOESM3_ESM.docx]

**Additional File 2**

| **Pathway** | **Overlap (Hits/Total Genes Involved)** | **Adjusted p-value** | **Genes** |
| --- | --- | --- | --- |
| Regulation Of RUNX1 Expression And Activity (R-HSA-8934593) | 3/17 | < 0,001 | CDK6,TNRC6A,TNRC6B |
| Oncogene Induced Senescence (R-HSA-2559585) | 3/33 | 0,035 | CDK6,TNRC6A,TNRC6B |
| Post-transcriptional Silencing By Small RNAs (R-HSA-426496) | 2/7 | 0,042 | TNRC6A,TNRC6B |
| Competing Endogenous RNAs (ceRNAs) Regulate PTEN Translation (R-HSA-8948700) | 2/10 | 0,052 | TNRC6A,TNRC6B |
| Regulation Of PTEN mRNA Translation (R-HSA-8943723) | 2/11 | 0,052 | TNRC6A,TNRC6B |
| Gene Expression (Transcription) (R-HSA-74160) | 10/1449 | 0,052 | BCL2L11,CDK6,CNOT1,SRSF1,BAZ2A,DICER1,JARID2,BDP1,TNRC6A,TNRC6B |
| Cell Cycle, Mitotic (R-HSA-69278) | 6/523 | 0,052 | WEE1,TUBA1A,CDK6,TUBB,TAOK1,ARPP19 |
| Oxidative Stress Induced Senescence (R-HSA-2559580) | 3/93 | 0,052 | CDK6,TNRC6A,TNRC6B |
| Post-chaperonin Tubulin Folding Pathway (R-HSA-389977) | 2/23 | 0,052 | TUBA1A,TUBB |
| Gene Silencing By RNA (R-HSA-211000) | 3/108 | 0,052 | DICER1,TNRC6A,TNRC6B |
| Formation Of Tubulin Folding Intermediates By CCT/TriC (R-HSA-389960) | 2/26 | 0,052 | TUBA1A,TUBB |
| Cell Cycle (R-HSA-1640170) | 6/654 | 0,052 | WEE1,TUBA1A,CDK6,TUBB,TAOK1,ARPP19 |
| Prefoldin Mediated Transfer Of Substrate To CCT/TriC (R-HSA-389957) | 2/28 | 0,052 | TUBA1A,TUBB |
| Regulation Of MECP2 Expression And Activity (R-HSA-9022692) | 2/31 | 0,052 | TNRC6A,TNRC6B |
| Sealing Of Nuclear Envelope (NE) By ESCRT-III (R-HSA-9668328) | 2/31 | 0,052 | TUBA1A,TUBB |
| Amino Acid Transport Across Plasma Membrane (R-HSA-352230) | 2/33 | 0,052 | SLC7A5,SLC38A2 |
| Cooperation Of Prefoldin And TriC/CCT In Actin And Tubulin Folding (R-HSA-389958) | 2/33 | 0,052 | TUBA1A,TUBB |
| NR1H3 And NR1H2 Regulate Gene Expression Linked To Cholesterol Transport And Efflux (R-HSA-9029569) | 2/36 | 0,052 | TNRC6A,TNRC6B |
| Transcriptional Regulation By VENTX (R-HSA-8853884) | 2/39 | 0,052 | TNRC6A,TNRC6B |
| Carboxyterminal Post-Translational Modifications Of Tubulin (R-HSA-8955332) | 2/41 | 0,052 | TUBA1A,TUBB |
| NR1H2 And NR1H3-mediated Signaling (R-HSA-9024446) | 2/46 | 0,052 | TNRC6A,TNRC6B |
| Cellular Senescence (R-HSA-2559583) | 3/165 | 0,052 | CDK6,TNRC6A,TNRC6B |
| G2/M Transition (R-HSA-69275) | 3/182 | 0,052 | WEE1,TUBA1A,TUBB |
| Mitotic G2-G2/M Phases (R-HSA-453274) | 3/184 | 0,052 | WEE1,TUBA1A,TUBB |
| Mitotic Prometaphase (R-HSA-68877) | 3/186 | 0,052 | TUBA1A,TUBB,TAOK1 |
| ESR-mediated Signaling (R-HSA-8939211) | 3/188 | 0,052 | TNRC6A,UHMK1,TNRC6B |
| M Phase (R-HSA-68886) | 4/380 | 0,052 | TUBA1A,TUBB,TAOK1,ARPP19 |
| Transcriptional Regulation By MECP2 (R-HSA-8986944) | 2/60 | 0,052 | TNRC6A,TNRC6B |
| Ca2+ Pathway (R-HSA-4086398) | 2/61 | 0,052 | TNRC6A,TNRC6B |
| Pre-NOTCH Transcription And Translation (R-HSA-1912408) | 2/62 | 0,052 | TNRC6A,TNRC6B |
| Transcriptional Regulation By RUNX1 (R-HSA-8878171) | 3/204 | 0,052 | CDK6,TNRC6A,TNRC6B |
| RUNX1 Regulates Genes Involved In Megakaryocyte Differentiation And Platelet Function (R-HSA-8936459) | 2/66 | 0,052 | TNRC6A,TNRC6B |
| Loss Of Nlp From Mitotic Centrosomes (R-HSA-380259) | 2/69 | 0,052 | TUBA1A,TUBB |
| AURKA Activation By TPX2 (R-HSA-8854518) | 2/72 | 0,053 | TUBA1A,TUBB |
| Nuclear Envelope (NE) Reassembly (R-HSA-2995410) | 2/75 | 0,053 | TUBA1A,TUBB |
| Mitotic Anaphase (R-HSA-68882) | 3/232 | 0,053 | TUBA1A,TUBB,TAOK1 |
| Mitotic Metaphase And Anaphase (R-HSA-2555396) | 3/233 | 0,053 | TUBA1A,TUBB,TAOK1 |
| Pre-NOTCH Expression And Processing (R-HSA-1912422) | 2/78 | 0,053 | TNRC6A,TNRC6B |
| G2/M DNA Replication Checkpoint (R-HSA-69478) | 1/5 | 0,053 | WEE1 |
| RUNX3 Regulates BCL2L11 (BIM) Transcription (R-HSA-8952158) | 1/5 | 0,053 | BCL2L11 |
| Drug-mediated Inhibition Of CDK4/CDK6 Activity (R-HSA-9754119) | 1/5 | 0,053 | CDK6 |
| Recruitment Of NuMA To Mitotic Centrosomes (R-HSA-380320) | 2/80 | 0,053 | TUBA1A,TUBB |
| TP53 Regulates Metabolic Genes (R-HSA-5628897) | 2/81 | 0,053 | TNRC6A,TNRC6B |
| Centrosome Maturation (R-HSA-380287) | 2/81 | 0,053 | TUBA1A,TUBB |
| Regulation Of PLK1 Activity At G2/M Transition (R-HSA-2565942) | 2/87 | 0,060 | TUBA1A,TUBB |
| Chaperonin-mediated Protein Folding (R-HSA-390466) | 2/90 | 0,060 | TUBA1A,TUBB |
| MAPK6/MAPK4 Signaling (R-HSA-5687128) | 2/90 | 0,060 | TNRC6A,TNRC6B |
| Signaling By Nuclear Receptors (R-HSA-9006931) | 3/260 | 0,060 | TNRC6A,UHMK1,TNRC6B |
| PIP3 Activates AKT Signaling (R-HSA-1257604) | 3/268 | 0,064 | FRS2,TNRC6A,TNRC6B |
| Protein Folding (R-HSA-391251) | 2/96 | 0,065 | TUBA1A,TUBB |
| Anchoring Of Basal Body To Plasma Membrane (R-HSA-5620912) | 2/97 | 0,065 | TUBA1A,TUBB |
| Transport Of Inorganic Cations/Anions And Amino Acids/Oligopeptides (R-HSA-425393) | 2/104 | 0,072 | SLC7A5,SLC38A2 |
| Small Interfering RNA (siRNA) Biogenesis (R-HSA-426486) | 1/9 | 0,072 | DICER1 |
| Activated NTRK2 Signals Thru FRS2 And FRS3 (R-HSA-9028731) | 1/9 | 0,072 | FRS2 |
| BH3-only Proteins Associate With And Inactivate Anti-Apoptotic BCL-2 Members (R-HSA-111453) | 1/9 | 0,072 | BCL2L11 |
| MASTL Facilitates Mitotic Progression (R-HSA-2465910) | 1/10 | 0,077 | ARPP19 |
| Signaling By FGFR4 In Disease (R-HSA-5655291) | 1/10 | 0,077 | FRS2 |
| Intracellular Signaling By Second Messengers (R-HSA-9006925) | 3/306 | 0,077 | FRS2,TNRC6A,TNRC6B |
| Epigenetic Regulation Of Gene Expression (R-HSA-212165) | 2/116 | 0,079 | BAZ2A,JARID2 |
| Endosomal/Vacuolar Pathway (R-HSA-1236977) | 1/11 | 0,080 | LNPEP |
| Estrogen-dependent Gene Expression (R-HSA-9018519) | 2/119 | 0,080 | TNRC6A,TNRC6B |
| MAPK Family Signaling Cascades (R-HSA-5683057) | 3/318 | 0,080 | FRS2,TNRC6A,TNRC6B |
| Frs2-mediated Activation (R-HSA-170968) | 1/12 | 0,083 | FRS2 |
| Synthesis Of PE (R-HSA-1483213) | 1/13 | 0,087 | ETNK1 |
| Chk1/Chk2(Cds1) Mediated Inactivation Of Cyclin B:Cdk1 Complex (R-HSA-75035) | 1/13 | 0,087 | WEE1 |
| Prolonged ERK Activation Events (R-HSA-169893) | 1/14 | 0,091 | FRS2 |
| Tryptophan Catabolism (R-HSA-71240) | 1/14 | 0,091 | SLC7A5 |
| PTEN Regulation (R-HSA-6807070) | 2/139 | 0,095 | TNRC6A,TNRC6B |
| Transcriptional Regulation By TP53 (R-HSA-3700989) | 3/354 | 0,095 | CNOT1,TNRC6A,TNRC6B |
| Beta-catenin Independent WNT Signaling (R-HSA-3858494) | 2/142 | 0,096 | TNRC6A,TNRC6B |
| FOXO-mediated Transcription Of Cell Death Genes (R-HSA-9614657) | 1/16 | 0,097 | BCL2L11 |
| Polo-like Kinase Mediated Events (R-HSA-156711) | 1/16 | 0,097 | WEE1 |
| Mitotic G1 Phase And G1/S Transition (R-HSA-453279) | 2/147 | 0,098 | WEE1,CDK6 |
| Aberrant Regulation Of Mitotic G1/S Transition In Cancer Due To RB1 Defects (R-HSA-9659787) | 1/17 | 0,100 | CDK6 |
| RNA Polymerase II Transcription (R-HSA-73857) | 6/1312 | 0,101 | BCL2L11,CDK6,CNOT1,SRSF1,TNRC6A,TNRC6B |
| PI-3K cascade:FGFR3 (R-HSA-5654710) | 1/18 | 0,103 | FRS2 |
| FRS-mediated FGFR3 Signaling (R-HSA-5654706) | 1/19 | 0,107 | FRS2 |
| PI-3K cascade:FGFR4 (R-HSA-5654720) | 1/20 | 0,109 | FRS2 |
| FRS-mediated FGFR4 Signaling (R-HSA-5654712) | 1/21 | 0,109 | FRS2 |
| PI-3K cascade:FGFR1 (R-HSA-5654689) | 1/21 | 0,109 | FRS2 |
| TP53 Regulates Transcription Of Additional Cell Cycle Genes With Uncertain Roles In P53 Pathway (R-HSA-6804115) | 1/21 | 0,109 | CNOT1 |
| Deregulated CDK5 Triggers Neurodegenerative Pathways In Alzheimers Disease Models (R-HSA-8862803) | 1/21 | 0,109 | BCL2L11 |
| Signaling By FGFR3 In Disease (R-HSA-56553329) | 1/21 | 0,109 | FRS2 |
| FRS-mediated FGFR1 Signaling (R-HSA-5654693) | 1/22 | 0,113 | FRS2 |
| PI-3K cascade:FGFR2 (R-HSA-5654695) | 1/23 | 0,113 | FRS2 |
| Glutamate Neurotransmitter Release Cycle (R-HSA-210500) | 1/23 | 0,113 | SLC38A2 |
| Signaling By NTRK2 (TRKB) (R-HSA-9006115) | 1/23 | 0,113 | FRS2 |
| FRS-mediated FGFR2 Signaling (R-HSA-5654700) | 1/24 | 0,113 | FRS2 |
| Defective Intrinsic Pathway For Apoptosis (R-HSA-9734009) | 1/24 | 0,113 | BCL2L11 |
| MicroRNA (miRNA) Biogenesis (R-HSA-203927) | 1/24 | 0,113 | DICER1 |
| Downstream Signaling Of Activated FGFR3 (R-HSA-5654708) | 1/24 | 0,113 | FRS2 |
| Estrogen-dependent Nuclear Events Downstream Of ESR-membrane Signaling (R-HSA-9634638) | 1/24 | 0,113 | UHMK1 |
| Basigin Interactions (R-HSA-210991) | 1/25 | 0,113 | SLC7A5 |
| Cyclin A/B1/B2 Associated Events During G2/M Transition (R-HSA-69273) | 1/25 | 0,113 | WEE1 |
| Deadenylation Of mRNA (R-HSA-429947) | 1/25 | 0,113 | CNOT1 |
| Cilium Assembly (R-HSA-5617833) | 2/186 | 0,113 | TUBA1A,TUBB |
| RNA Polymerase III Transcription Initiation From Type 2 Promoter (R-HSA-76066) | 1/26 | 0,113 | BDP1 |
| Downstream Signaling Of Activated FGFR4 (R-HSA-5654716) | 1/26 | 0,113 | FRS2 |
| Signaling By ALK (R-HSA-201556) | 1/26 | 0,113 | FRS2 |
| RNA Polymerase III Transcription Initiation From Type 1 Promoter (R-HSA-76061) | 1/27 | 0,115 | BDP1 |
| RNA Polymerase III Transcription Initiation From Type 3 Promoter (R-HSA-76071) | 1/27 | 0,115 | BDP1 |
| Downstream Signaling Of Activated FGFR2 (R-HSA-5654696) | 1/29 | 0,121 | FRS2 |
| Negative Regulation Of FGFR3 Signaling (R-HSA-5654732) | 1/29 | 0,121 | FRS2 |
| Activation Of BH3-only Proteins (R-HSA-114452) | 1/30 | 0,122 | BCL2L11 |
| Downstream Signaling Of Activated FGFR1 (R-HSA-5654687) | 1/30 | 0,122 | FRS2 |
| Signaling By NOTCH (R-HSA-157118) | 2/203 | 0,122 | TNRC6A,TNRC6B |
| Negative Regulation Of FGFR4 Signaling (R-HSA-5654733) | 1/31 | 0,124 | FRS2 |
| Signaling To ERKs (R-HSA-187687) | 1/33 | 0,130 | FRS2 |
| Negative Regulation Of FGFR1 Signaling (R-HSA-5654726) | 1/33 | 0,130 | FRS2 |
| Negative Regulation Of FGFR2 Signaling (R-HSA-5654727) | 1/34 | 0,132 | FRS2 |
| RNA Polymerase III Transcription Initiation (R-HSA-76046) | 1/35 | 0,135 | BDP1 |
| Aberrant Regulation Of Mitotic Cell Cycle Due To RB1 Defects (R-HSA-9687139) | 1/36 | 0,136 | CDK6 |
| Signaling By FGFR1 In Disease (R-HSA-5655302) | 1/36 | 0,136 | FRS2 |
| FLT3 Signaling (R-HSA-9607240) | 1/37 | 0,138 | BCL2L11 |
| Generic Transcription Pathway (R-HSA-212436) | 5/1190 | 0,138 | BCL2L11,CDK6,CNOT1,TNRC6A,TNRC6B |
| Diseases Of Mitotic Cell Cycle (R-HSA-9675126) | 1/38 | 0,140 | CDK6 |
| Signaling By FGFR3 (R-HSA-5654741) | 1/39 | 0,142 | FRS2 |
| RET Signaling (R-HSA-8853659) | 1/40 | 0,142 | FRS2 |
| RNA Polymerase III Abortive And Retractive Initiation (R-HSA-749476) | 1/40 | 0,142 | BDP1 |
| Signaling By FGFR4 (R-HSA-5654743) | 1/40 | 0,142 | FRS2 |
| PRC2 Methylates Histones And DNA (R-HSA-212300) | 1/42 | 0,145 | JARID2 |
| RND1 GTPase Cycle (R-HSA-9696273) | 1/42 | 0,145 | FRS2 |
| Signaling By FGFR2 In Disease (R-HSA-5655253) | 1/42 | 0,145 | FRS2 |
| SLC-mediated Transmembrane Transport (R-HSA-425407) | 2/247 | 0,146 | SLC7A5,SLC38A2 |
| RND2 GTPase Cycle (R-HSA-9696270) | 1/43 | 0,146 | FRS2 |
| PI3K Cascade (R-HSA-109704) | 1/44 | 0,148 | FRS2 |
| IRS-mediated Signaling (R-HSA-112399) | 1/47 | 0,155 | FRS2 |
| Cyclin D Associated Events In G1 (R-HSA-69231) | 1/47 | 0,155 | CDK6 |
| TP53 Regulates Transcription Of Cell Cycle Genes (R-HSA-6791312) | 1/49 | 0,158 | CNOT1 |
| mRNA Splicing - Minor Pathway (R-HSA-72165) | 1/49 | 0,158 | SRSF1 |
| Signaling By FGFR1 (R-HSA-5654736) | 1/49 | 0,158 | FRS2 |
| Neurotransmitter Release Cycle (R-HSA-112310) | 1/50 | 0,159 | SLC38A2 |
| Cell Cycle Checkpoints (R-HSA-69620) | 2/271 | 0,159 | WEE1,TAOK1 |
| Translocation Of SLC2A4 (GLUT4) To Plasma Membrane (R-HSA-1445148) | 1/51 | 0,159 | LNPEP |
| IRS-related Events Triggered By IGF1R (R-HSA-2428928) | 1/51 | 0,159 | FRS2 |
| Organelle Biogenesis And Maintenance (R-HSA-1852241) | 2/275 | 0,160 | TUBA1A,TUBB |
| IGF1R Signaling Cascade (R-HSA-2428924) | 1/52 | 0,160 | FRS2 |
| Insulin Receptor Signaling Cascade (R-HSA-74751) | 1/53 | 0,160 | FRS2 |
| Signaling By Type 1 Insulin-like Growth Factor 1 Receptor (IGF1R) (R-HSA-2404192) | 1/53 | 0,160 | FRS2 |
| Intrinsic Pathway For Apoptosis (R-HSA-109606) | 1/55 | 0,163 | BCL2L11 |
| Regulation Of Cholesterol Biosynthesis By SREBP (SREBF) (R-HSA-1655829) | 1/55 | 0,163 | INSIG1 |
| Signaling By ALK Fusions And Activated Point Mutants (R-HSA-9725370) | 1/55 | 0,163 | FRS2 |
| Deadenylation-dependent mRNA Decay (R-HSA-429914) | 1/56 | 0,164 | CNOT1 |
| NRAGE Signals Death Thru JNK (R-HSA-193648) | 1/57 | 0,166 | BCL2L11 |
| mRNA 3-End Processing (R-HSA-72187) | 1/58 | 0,167 | SRSF1 |
| Signaling By WNT (R-HSA-195721) | 2/294 | 0,167 | TNRC6A,TNRC6B |
| Signaling By FGFR In Disease (R-HSA-1226099) | 1/61 | 0,173 | FRS2 |
| Signaling By BRAF And RAF1 Fusions (R-HSA-6802952) | 1/62 | 0,175 | BCL2L11 |
| FOXO-mediated Transcription (R-HSA-9614085) | 1/65 | 0,181 | BCL2L11 |
| RNA Polymerase II Transcription Termination (R-HSA-73856) | 1/67 | 0,185 | SRSF1 |
| Diseases Of Programmed Cell Death (R-HSA-9645723) | 1/71 | 0,194 | BCL2L11 |
| Signaling By FGFR2 (R-HSA-5654738) | 1/72 | 0,196 | FRS2 |
| Extra-nuclear Estrogen Signaling (R-HSA-9009391) | 1/73 | 0,196 | UHMK1 |
| Transport Of Mature mRNA Derived From An Intron-Containing Transcript (R-HSA-159236) | 1/74 | 0,196 | SRSF1 |
| Cell Death Signaling Via NRAGE, NRIF And NADE (R-HSA-204998) | 1/74 | 0,196 | BCL2L11 |
| NoRC Negatively Regulates rRNA Expression (R-HSA-427413) | 1/74 | 0,196 | BAZ2A |
| Transcriptional Regulation By Small RNAs (R-HSA-5578749) | 1/76 | 0,197 | TNRC6A |
| G2/M DNA Damage Checkpoint (R-HSA-69473) | 1/77 | 0,197 | WEE1 |
| Negative Epigenetic Regulation Of rRNA Expression (R-HSA-5250941) | 1/77 | 0,197 | BAZ2A |
| Signaling By Insulin Receptor (R-HSA-74752) | 1/77 | 0,197 | FRS2 |
| Oncogenic MAPK Signaling (R-HSA-6802957) | 1/78 | 0,197 | BCL2L11 |
| COPI-dependent Golgi-to-ER Retrograde Traffic (R-HSA-6811434) | 1/78 | 0,197 | SURF4 |
| Constitutive Signaling By Aberrant PI3K In Cancer (R-HSA-2219530) | 1/78 | 0,197 | FRS2 |
| Senescence-Associated Secretory Phenotype (SASP) (R-HSA-2559582) | 1/81 | 0,203 | CDK6 |
| Cyclin E Associated Events During G1/S Transition (R-HSA-69202) | 1/82 | 0,204 | WEE1 |
| Transport Of Mature Transcript To Cytoplasm (R-HSA-72202) | 1/83 | 0,205 | SRSF1 |
| Cyclin A:Cdk2-associated Events At S Phase Entry (R-HSA-69656) | 1/84 | 0,206 | WEE1 |
| Signaling By FGFR (R-HSA-190236) | 1/86 | 0,209 | FRS2 |
| Cellular Responses To Stress (R-HSA-2262752) | 3/722 | 0,209 | CDK6,TNRC6A,TNRC6B |
| Cellular Responses To Stimuli (R-HSA-8953897) | 3/736 | 0,216 | CDK6,TNRC6A,TNRC6B |
| Unattached Kinetochores Signal Amplification Via A MAD2 Inhibitory Signal (R-HSA-141444) | 1/93 | 0,221 | TAOK1 |
| Transcriptional Regulation By RUNX3 (R-HSA-8878159) | 1/95 | 0,222 | BCL2L11 |
| P75 NTR Receptor-Mediated Signaling (R-HSA-193704) | 1/95 | 0,222 | BCL2L11 |
| Potential Therapeutics For SARS (R-HSA-9679191) | 1/97 | 0,224 | TUBB |
| EML4 And NUDC In Mitotic Spindle Formation (R-HSA-9648025) | 1/97 | 0,224 | TAOK1 |
| Antigen processing-Cross Presentation (R-HSA-1236975) | 1/104 | 0,237 | LNPEP |
| PI3K/AKT Signaling In Cancer (R-HSA-2219528) | 1/105 | 0,238 | FRS2 |
| PI5P, PP2A And IER3 Regulate PI3K/AKT Signaling (R-HSA-6811558) | 1/106 | 0,238 | FRS2 |
| Resolution Of Sister Chromatid Cohesion (R-HSA-2500257) | 1/106 | 0,238 | TAOK1 |
| Interleukin-4 And Interleukin-13 Signaling (R-HSA-6785807) | 1/107 | 0,238 | MCL1 |
| Diseases Of Signal Transduction By Growth Factor Receptors And Second Messengers (R-HSA-5663202) | 2/424 | 0,242 | BCL2L11,FRS2 |
| Mitotic Spindle Checkpoint (R-HSA-69618) | 1/110 | 0,242 | TAOK1 |
| Golgi-to-ER Retrograde Transport (R-HSA-8856688) | 1/112 | 0,243 | SURF4 |
| Mitotic Prophase (R-HSA-68875) | 1/112 | 0,243 | ARPP19 |
| Negative Regulation Of PI3K/AKT Network (R-HSA-199418) | 1/113 | 0,244 | FRS2 |
| Signaling By NTRK1 (TRKA) (R-HSA-187037) | 1/114 | 0,244 | FRS2 |
| RHO GTPases Activate Formins (R-HSA-5663220) | 1/119 | 0,252 | TAOK1 |
| Glycerophospholipid Biosynthesis (R-HSA-1483206) | 1/127 | 0,266 | ETNK1 |
| G1/S Transition (R-HSA-69206) | 1/129 | 0,268 | WEE1 |
| Neutrophil Degranulation (R-HSA-6798695) | 2/468 | 0,269 | TUBB,SURF4 |
| Signaling By NTRKs (R-HSA-166520) | 1/132 | 0,271 | FRS2 |
| Cell Surface Interactions At Vascular Wall (R-HSA-202733) | 1/134 | 0,273 | SLC7A5 |
| Factors Involved In Megakaryocyte Development And Platelet Production (R-HSA-983231) | 1/136 | 0,275 | WEE1 |
| Death Receptor Signaling (R-HSA-73887) | 1/139 | 0,279 | BCL2L11 |
| G2/M Checkpoints (R-HSA-69481) | 1/148 | 0,294 | WEE1 |
| Metabolism Of Steroids (R-HSA-8957322) | 1/153 | 0,301 | INSIG1 |
| S Phase (R-HSA-69242) | 1/161 | 0,313 | WEE1 |
| Separation Of Sister Chromatids (R-HSA-2467813) | 1/170 | 0,326 | TAOK1 |
| Apoptosis (R-HSA-109581) | 1/178 | 0,337 | BCL2L11 |
| mRNA Splicing - Major Pathway (R-HSA-72163) | 1/181 | 0,338 | SRSF1 |
| Intra-Golgi And Retrograde Golgi-to-ER Traffic (R-HSA-6811442) | 1/181 | 0,338 | SURF4 |
| Immune System (R-HSA-168256) | 5/1943 | 0,338 | BCL2L11,TUBB,SURF4,LNPEP,MCL1 |
| Hemostasis (R-HSA-109582) | 2/576 | 0,338 | SLC7A5,WEE1 |
| mRNA Splicing (R-HSA-72172) | 1/189 | 0,346 | SRSF1 |
| Signal Transduction (R-HSA-162582) | 6/2465 | 0,350 | BCL2L11,TAOK1,FRS2,TNRC6A,UHMK1,TNRC6B |
| Membrane Trafficking (R-HSA-199991) | 2/599 | 0,352 | SURF4,LNPEP |
| Phospholipid Metabolism (R-HSA-1483257) | 1/208 | 0,367 | ETNK1 |
| Programmed Cell Death (R-HSA-5357801) | 1/208 | 0,367 | BCL2L11 |
| Vesicle-mediated Transport (R-HSA-5653656) | 2/637 | 0,377 | SURF4,LNPEP |
| Signaling By Rho GTPases (R-HSA-194315) | 2/644 | 0,380 | TAOK1,FRS2 |
| Signaling By Rho GTPases, Miro GTPases And RHOBTB3 (R-HSA-9716542) | 2/660 | 0,391 | TAOK1,FRS2 |
| Metabolism Of RNA (R-HSA-8953854) | 2/666 | 0,393 | CNOT1,SRSF1 |
| Processing Of Capped Intron-Containing Pre-mRNA (R-HSA-72203) | 1/242 | 0,406 | SRSF1 |
| Transmission Across Chemical Synapses (R-HSA-112315) | 1/246 | 0,409 | SLC38A2 |
| Cytokine Signaling In Immune System (R-HSA-1280215) | 2/702 | 0,415 | BCL2L11,MCL1 |
| Transport Of Small Molecules (R-HSA-382551) | 2/706 | 0,416 | SLC7A5,SLC38A2 |
| RHO GTPase Effectors (R-HSA-195258) | 1/269 | 0,431 | TAOK1 |
| Metabolism Of Lipids (R-HSA-556833) | 2/732 | 0,431 | INSIG1,ETNK1 |
| RAF/MAP Kinase Cascade (R-HSA-5673001) | 1/271 | 0,431 | FRS2 |
| Disease (R-HSA-1643685) | 4/1736 | 0,434 | BCL2L11,CDK6,TUBB,FRS2 |
| MAPK1/MAPK3 Signaling (R-HSA-5684996) | 1/277 | 0,434 | FRS2 |
| Antigen Processing: Ubiquitination And Proteasome Degradation (R-HSA-983168) | 1/307 | 0,468 | LNPEP |
| Metabolism Of Amino Acids And Derivatives (R-HSA-71291) | 1/364 | 0,527 | SLC7A5 |
| SARS-CoV Infections (R-HSA-9679506) | 1/369 | 0,529 | TUBB |
| Class I MHC Mediated Antigen Processing And Presentation (R-HSA-983169) | 1/378 | 0,536 | LNPEP |
| Neuronal System (R-HSA-112316) | 1/386 | 0,541 | SLC38A2 |
| RHO GTPase Cycle (R-HSA-9012999) | 1/441 | 0,589 | FRS2 |
| Signaling By Interleukins (R-HSA-449147) | 1/453 | 0,597 | MCL1 |
| Innate Immune System (R-HSA-168249) | 2/1035 | 0,600 | TUBB,SURF4 |
| Signaling By Receptor Tyrosine Kinases (R-HSA-9006934) | 1/496 | 0,627 | FRS2 |
| Axon Guidance (R-HSA-422475) | 1/519 | 0,641 | FRS2 |
| Nervous System Development (R-HSA-9675108) | 1/545 | 0,657 | FRS2 |
| Post-translational Protein Modification (R-HSA-597592) | 2/1383 | 0,752 | TUBA1A,TUBB |
| Metabolism (R-HSA-1430728) | 3/2049 | 0,758 | SLC7A5,INSIG1,ETNK1 |
| Adaptive Immune System (R-HSA-1280218) | 1/733 | 0,759 | LNPEP |
| Infectious Disease (R-HSA-5663205) | 1/961 | 0,846 | TUBB |
| Developmental Biology (R-HSA-1266738) | 1/1073 | 0,874 | FRS2 |
| Metabolism Of Proteins (R-HSA-392499) | 2/1890 | 0,877 | TUBA1A,TUBB |
