# Supplementary material for: In silico analysis of prognostic and diagnostic significance of target genes from prostate cancer cell lines derived exomicroRNAs
Source: Cancer Cell Int. 2023 Nov 17;23:275. doi: 10.1186/s12935-023-03123-1 (PMC10655318; doi:10.1186/s12935-023-03123-1)
Supplement: Supplementary file 4 — Additional file 4. Full Reactome pathway enrichment analysis list of pathways affected by upregulated exomiRNAs in PC-3 cells vs LNCaP cell lines and putative implicated target genes. [file 12935_2023_3123_MOESM4_ESM.docx]

Additional File 3

| Pathway | Hits/Total Genes Involved | Adjusted p-value | Genes |
| --- | --- | --- | --- |
| Regulation Of RUNX1 Expression And Activity (R-HSA-8934593) | 6/17 | < 0.001 | CDK6,AGO3,CCND1,AGO1,TNRC6A,TNRC6B |
| Post-transcriptional Silencing By Small RNAs (R-HSA-426496) | 4/7 | < 0.001 | AGO3,AGO1,TNRC6A,TNRC6B |
| Competing Endogenous RNAs (ceRNAs) Regulate PTEN Translation (R-HSA-8948700) | 4/10 | < 0.001 | AGO3,AGO1,TNRC6A,TNRC6B |
| RUNX1 Regulates Genes Involved In Megakaryocyte Differentiation And Platelet Function (R-HSA-8936459) | 6/66 | < 0.001 | KMT2D,AGO3,AGO1,THBS1,TNRC6A,TNRC6B |
| Regulation Of PTEN mRNA Translation (R-HSA-8943723) | 4/11 | < 0.001 | AGO3,AGO1,TNRC6A,TNRC6B |
| Oncogene Induced Senescence (R-HSA-2559585) | 5/33 | < 0.001 | CDK6,AGO3,AGO1,TNRC6A,TNRC6B |
| Transcriptional Regulation By RUNX1 (R-HSA-8878171) | 8/204 | < 0.001 | KMT2D,CDK6,CCND1,AGO3,AGO1,THBS1,TNRC6A,TNRC6B |
| Transcriptional Regulation By VENTX (R-HSA-8853884) | 5/39 | < 0.001 | CCND1,AGO3,AGO1,TNRC6A,TNRC6B |
| TP53 Regulates Metabolic Genes (R-HSA-5628897) | 6/81 | < 0.001 | SESN3,AGO3,AGO1,YWHAG,TNRC6A,TNRC6B |
| Gene Expression (Transcription) (R-HSA-74160) | 16/1449 | < 0.001 | KMT2D,BTG2,DICER1,THBS1,CDK6,CCND1,SESN3,AGO3,AGO1,TP53INP1,TET3,PMAIP1,ZNF711,YWHAG,TNRC6A,TNRC6B |
| Transcriptional Regulation By TP53 (R-HSA-3700989) | 9/354 | < 0.001 | BTG2,SESN3,AGO3,AGO1,TP53INP1,PMAIP1,YWHAG,TNRC6A,TNRC6B |
| Generic Transcription Pathway (R-HSA-212436) | 14/1190 | < 0.001 | KMT2D,BTG2,THBS1,CDK6,CCND1,SESN3,AGO3,AGO1,TP53INP1,PMAIP1,ZNF711,YWHAG,TNRC6A,TNRC6B |
| ESR-mediated Signaling (R-HSA-8939211) | 7/188 | < 0.001 | CCND1,AGO3,AGO1,ZNF217,TNRC6A,UHMK1,TNRC6B |
| Estrogen-dependent Gene Expression (R-HSA-9018519) | 6/119 | < 0.001 | CCND1,AGO3,AGO1,ZNF217,TNRC6A,TNRC6B |
| Pre-NOTCH Transcription And Translation (R-HSA-1912408) | 5/62 | < 0.001 | CCND1,AGO3,AGO1,TNRC6A,TNRC6B |
| RNA Polymerase II Transcription (R-HSA-73857) | 14/1312 | < 0.001 | KMT2D,BTG2,THBS1,CDK6,CCND1,SESN3,AGO3,AGO1,TP53INP1,PMAIP1,ZNF711,YWHAG,TNRC6A,TNRC6B |
| Regulation Of MECP2 Expression And Activity (R-HSA-9022692) | 4/31 | < 0.001 | AGO3,AGO1,TNRC6A,TNRC6B |
| Pre-NOTCH Expression And Processing (R-HSA-1912422) | 5/78 | < 0.001 | CCND1,AGO3,AGO1,TNRC6A,TNRC6B |
| Small Interfering RNA (siRNA) Biogenesis (R-HSA-426486) | 3/9 | < 0.001 | AGO3,AGO1,DICER1 |
| NR1H3 And NR1H2 Regulate Gene Expression Linked To Cholesterol Transport And Efflux (R-HSA-9029569) | 4/36 | < 0.001 | AGO3,AGO1,TNRC6A,TNRC6B |
| Signaling By Nuclear Receptors (R-HSA-9006931) | 7/260 | < 0.001 | CCND1,AGO3,AGO1,ZNF217,TNRC6A,UHMK1,TNRC6B |
| Oxidative Stress Induced Senescence (R-HSA-2559580) | 5/93 | < 0.001 | CDK6,AGO3,AGO1,TNRC6A,TNRC6B |
| NR1H2 And NR1H3-mediated Signaling (R-HSA-9024446) | 4/46 | < 0.001 | AGO3,AGO1,TNRC6A,TNRC6B |
| Gene Silencing By RNA (R-HSA-211000) | 5/108 | < 0.001 | AGO3,AGO1,DICER1,TNRC6A,TNRC6B |
| Transcriptional Regulation By MECP2 (R-HSA-8986944) | 4/60 | < 0.001 | AGO3,AGO1,TNRC6A,TNRC6B |
| Ca2+ Pathway (R-HSA-4086398) | 4/61 | < 0.001 | AGO3,AGO1,TNRC6A,TNRC6B |
| Beta-catenin Independent WNT Signaling (R-HSA-3858494) | 5/142 | < 0.001 | AGO3,AGO1,CLTC,TNRC6A,TNRC6B |
| MicroRNA (miRNA) Biogenesis (R-HSA-203927) | 3/24 | < 0.001 | AGO3,AGO1,DICER1 |
| Cellular Senescence (R-HSA-2559583) | 5/165 | < 0.001 | CDK6,AGO3,AGO1,TNRC6A,TNRC6B |
| Signaling By WNT (R-HSA-195721) | 6/294 | < 0.001 | KMT2D,AGO3,AGO1,CLTC,TNRC6A,TNRC6B |
| MAPK6/MAPK4 Signaling (R-HSA-5687128) | 4/90 | < 0.001 | AGO3,AGO1,TNRC6A,TNRC6B |
| Drug-mediated Inhibition Of CDK4/CDK6 Activity (R-HSA-9754119) | 2/5 | < 0.001 | CDK6,CCND1 |
| Signaling By NOTCH (R-HSA-157118) | 5/203 | < 0.001 | CCND1,AGO3,AGO1,TNRC6A,TNRC6B |
| PTEN Regulation (R-HSA-6807070) | 4/139 | 0,001 | AGO3,AGO1,TNRC6A,TNRC6B |
| Chk1/Chk2(Cds1) Mediated Inactivation Of Cyclin B:Cdk1 Complex (R-HSA-75035) | 2/13 | 0,002 | WEE1,YWHAG |
| Signal Transduction (R-HSA-162582) | 14/2465 | 0,003 | KMT2D,BMPR2,CLTC,LAMC1,THBS1,UHMK1,CCND1,AGO3,TAOK1,AGO1,ZNF217,YWHAG,TNRC6A,TNRC6B |
| Aberrant Regulation Of Mitotic G1/S Transition In Cancer Due To RB1 Defects (R-HSA-9659787) | 2/17 | 0,003 | CDK6,CCND1 |
| TP53 Regulates Transcription Of Genes Involved In Cytochrome C Release (R-HSA-6803204) | 2/20 | 0,005 | TP53INP1,PMAIP1 |
| Estrogen-dependent Nuclear Events Downstream Of ESR-membrane Signaling (R-HSA-9634638) | 2/24 | 0,007 | CCND1,UHMK1 |
| Chromatin Modifying Enzymes (R-HSA-3247509) | 4/238 | 0,009 | KMT2D,CCND1,ASH1L,BRWD1 |
| Activation Of BH3-only Proteins (R-HSA-114452) | 2/30 | 0,010 | PMAIP1,YWHAG |
| PIP3 Activates AKT Signaling (R-HSA-1257604) | 4/268 | 0,013 | AGO3,AGO1,TNRC6A,TNRC6B |
| Aberrant Regulation Of Mitotic Cell Cycle Due To RB1 Defects (R-HSA-9687139) | 2/36 | 0,014 | CDK6,CCND1 |
| Diseases Of Mitotic Cell Cycle (R-HSA-9675126) | 2/38 | 0,015 | CDK6,CCND1 |
| Non-integrin membrane-ECM Interactions (R-HSA-3000171) | 2/41 | 0,017 | LAMC1,THBS1 |
| Mitotic G1 Phase And G1/S Transition (R-HSA-453279) | 3/147 | 0,018 | WEE1,CDK6,CCND1 |
| TP53 Regulates Transcription Of Cell Death Genes (R-HSA-5633008) | 2/44 | 0,018 | TP53INP1,PMAIP1 |
| Intracellular Signaling By Second Messengers (R-HSA-9006925) | 4/306 | 0,018 | AGO3,AGO1,TNRC6A,TNRC6B |
| PKMTs Methylate Histone Lysines (R-HSA-3214841) | 2/47 | 0,020 | KMT2D,ASH1L |
| Cyclin D Associated Events In G1 (R-HSA-69231) | 2/47 | 0,020 | CDK6,CCND1 |
| MAPK Family Signaling Cascades (R-HSA-5683057) | 4/318 | 0,020 | AGO3,AGO1,TNRC6A,TNRC6B |
| Cell Cycle, Mitotic (R-HSA-69278) | 5/523 | 0,021 | WEE1,CDK6,CCND1,TAOK1,YWHAG |
| Intrinsic Pathway For Apoptosis (R-HSA-109606) | 2/55 | 0,025 | PMAIP1,YWHAG |
| SARS-CoV-2-host Interactions (R-HSA-9705683) | 3/196 | 0,034 | LARP1,G3BP1,YWHAG |
| Sodium-coupled Phosphate Cotransporters (R-HSA-427652) | 1/5 | 0,040 | SLC20A1 |
| Activation Of NOXA And Translocation To Mitochondria (R-HSA-111448) | 1/5 | 0,040 | PMAIP1 |
| G2/M DNA Replication Checkpoint (R-HSA-69478) | 1/5 | 0,040 | WEE1 |
| Extra-nuclear Estrogen Signaling (R-HSA-9009391) | 2/73 | 0,040 | CCND1,UHMK1 |
| Transcriptional Regulation By Small RNAs (R-HSA-5578749) | 2/76 | 0,042 | AGO1,TNRC6A |
| G2/M DNA Damage Checkpoint (R-HSA-69473) | 2/77 | 0,043 | WEE1,YWHAG |
| Cell Cycle (R-HSA-1640170) | 5/654 | 0,044 | WEE1,CDK6,CCND1,TAOK1,YWHAG |
| PTK6 Regulates Cell Cycle (R-HSA-8849470) | 1/6 | 0,044 | CCND1 |
| Entry Of Influenza Virion Into Host Cell Via Endocytosis (R-HSA-168275) | 1/6 | 0,044 | CLTC |
| Cyclin E Associated Events During G1/S Transition (R-HSA-69202) | 2/82 | 0,045 | WEE1,CCND1 |
| Cyclin A:Cdk2-associated Events At S Phase Entry (R-HSA-69656) | 2/84 | 0,046 | WEE1,CCND1 |
| RUNX3 Regulates WNT Signaling (R-HSA-8951430) | 1/8 | 0,055 | CCND1 |
| Cellular Responses To Stress (R-HSA-2262752) | 5/722 | 0,059 | CDK6,AGO3,AGO1,TNRC6A,TNRC6B |
| Formation Of Annular Gap Junctions (R-HSA-196025) | 1/9 | 0,059 | CLTC |
| BH3-only Proteins Associate With And Inactivate Anti-Apoptotic BCL-2 Members (R-HSA-111453) | 1/9 | 0,059 | PMAIP1 |
| L1CAM Interactions (R-HSA-373760) | 2/99 | 0,059 | CLTC,LAMC1 |
| Cellular Responses To Stimuli (R-HSA-8953897) | 5/736 | 0,061 | CDK6,AGO3,AGO1,TNRC6A,TNRC6B |
| Cell Cycle Checkpoints (R-HSA-69620) | 3/271 | 0,061 | WEE1,TAOK1,YWHAG |
| Gap Junction Degradation (R-HSA-190873) | 1/10 | 0,062 | CLTC |
| RUNX3 Regulates p14-ARF (R-HSA-8951936) | 1/10 | 0,062 | CCND1 |
| SARS-CoV-2 Infection (R-HSA-9694516) | 3/283 | 0,065 | LARP1,G3BP1,YWHAG |
| WNT5A-dependent Internalization Of FZD2, FZD5 And ROR2 (R-HSA-5140745) | 1/11 | 0,066 | CLTC |
| ALK Mutants Bind TKIs (R-HSA-9700645) | 1/12 | 0,070 | CLTC |
| Regulation Of Localization Of FOXO Transcription Factors (R-HSA-9614399) | 1/12 | 0,070 | YWHAG |
| SARS-CoV-2 Targets Host Intracellular Signaling And Regulatory Pathways (R-HSA-9755779) | 1/13 | 0,075 | YWHAG |
| SARS-CoV-2 Activates/Modulates Innate And Adaptive Immune Responses (R-HSA-9705671) | 2/121 | 0,075 | LARP1,G3BP1 |
| Retrograde Neurotrophin Signaling (R-HSA-177504) | 1/14 | 0,078 | CLTC |
| G1/S Transition (R-HSA-69206) | 2/129 | 0,081 | WEE1,CCND1 |
| Activation Of BAD And Translocation To Mitochondria (R-HSA-111447) | 1/15 | 0,081 | YWHAG |
| WNT5A-dependent Internalization Of FZD4 (R-HSA-5099900) | 1/15 | 0,081 | CLTC |
| Polo-like Kinase Mediated Events (R-HSA-156711) | 1/16 | 0,084 | WEE1 |
| VLDLR Internalisation And Degradation (R-HSA-8866427) | 1/16 | 0,084 | CLTC |
| MET Activates PTK2 Signaling (R-HSA-8874081) | 1/17 | 0,088 | LAMC1 |
| LDL Clearance (R-HSA-8964038) | 1/18 | 0,092 | CLTC |
| Nuclear Events Stimulated By ALK Signaling In Cancer (R-HSA-9725371) | 1/19 | 0,096 | CLTC |
| G2/M Checkpoints (R-HSA-69481) | 2/148 | 0,096 | WEE1,YWHAG |
| Syndecan Interactions (R-HSA-3000170) | 1/20 | 0,099 | THBS1 |
| TP53 Regulates Transcription Of Additional Cell Cycle Genes With Uncertain Roles In P53 Pathway (R-HSA-6804115) | 1/21 | 0,103 | BTG2 |
| SARS-CoV Infections (R-HSA-9679506) | 3/369 | 0,103 | LARP1,G3BP1,YWHAG |
| Laminin Interactions (R-HSA-3000157) | 1/22 | 0,105 | LAMC1 |
| S Phase (R-HSA-69242) | 2/161 | 0,106 | WEE1,CCND1 |
| Gap Junction Trafficking (R-HSA-190828) | 1/25 | 0,114 | CLTC |
| Interleukin-7 Signaling (R-HSA-1266695) | 1/25 | 0,114 | BRWD1 |
| Cyclin A/B1/B2 Associated Events During G2/M Transition (R-HSA-69273) | 1/25 | 0,114 | WEE1 |
| Apoptosis (R-HSA-109581) | 2/178 | 0,120 | PMAIP1,YWHAG |
| Gap Junction Trafficking And Regulation (R-HSA-157858) | 1/27 | 0,120 | CLTC |
| Signaling By BMP (R-HSA-201451) | 1/27 | 0,120 | BMPR2 |
| G2/M Transition (R-HSA-69275) | 2/182 | 0,120 | WEE1,YWHAG |
| Recycling Pathway Of L1 (R-HSA-437239) | 1/28 | 0,120 | CLTC |
| MET Promotes Cell Motility (R-HSA-8875878) | 1/28 | 0,120 | LAMC1 |
| Mitotic G2-G2/M Phases (R-HSA-453274) | 2/184 | 0,121 | WEE1,YWHAG |
| EGR2 And SOX10-mediated Initiation Of Schwann Cell Myelination (R-HSA-9619665) | 1/29 | 0,121 | LAMC1 |
| Mitotic Prometaphase (R-HSA-68877) | 2/186 | 0,121 | TAOK1,YWHAG |
| Lysosome Vesicle Biogenesis (R-HSA-432720) | 1/34 | 0,140 | CLTC |
| Plasma Lipoprotein Clearance (R-HSA-8964043) | 1/35 | 0,143 | CLTC |
| Programmed Cell Death (R-HSA-5357801) | 2/208 | 0,143 | PMAIP1,YWHAG |
| Disease (R-HSA-1643685) | 7/1736 | 0,145 | LARP1,CDK6,CCND1,CLTC,G3BP1,THBS1,YWHAG |
| RHOV GTPase Cycle (R-HSA-9013424) | 1/37 | 0,145 | CLTC |
| Defective B3GALTL Causes PpS (R-HSA-5083635) | 1/37 | 0,145 | THBS1 |
| O-glycosylation Of TSR Domain-Containing Proteins (R-HSA-5173214) | 1/38 | 0,148 | THBS1 |
| Ovarian Tumor Domain Proteases (R-HSA-5689896) | 1/39 | 0,149 | YOD1 |
| RHOU GTPase Cycle (R-HSA-9013420) | 1/39 | 0,149 | CLTC |
| Deactivation Of Beta-Catenin Transactivating Complex (R-HSA-3769402) | 1/42 | 0,158 | KMT2D |
| Signaling By Receptor Tyrosine Kinases (R-HSA-9006934) | 3/496 | 0,164 | CLTC,LAMC1,THBS1 |
| TP53 Regulates Transcription Of Cell Cycle Genes (R-HSA-6791312) | 1/49 | 0,177 | BTG2 |
| RMTs Methylate Histone Arginines (R-HSA-3214858) | 1/49 | 0,177 | CCND1 |
| EPH-ephrin Mediated Repulsion Of Cells (R-HSA-3928665) | 1/49 | 0,177 | CLTC |
| Translocation Of SLC2A4 (GLUT4) To Plasma Membrane (R-HSA-1445148) | 1/51 | 0,181 | YWHAG |
| Ubiquitin-dependent Degradation Of Cyclin D (R-HSA-75815) | 1/51 | 0,181 | CCND1 |
| Signaling By Non-Receptor Tyrosine Kinases (R-HSA-9006927) | 1/52 | 0,181 | CCND1 |
| Signaling By PDGF (R-HSA-186797) | 1/52 | 0,181 | THBS1 |
| Golgi Associated Vesicle Biogenesis (R-HSA-432722) | 1/55 | 0,187 | CLTC |
| Signaling By ALK Fusions And Activated Point Mutants (R-HSA-9725370) | 1/55 | 0,187 | CLTC |
| ECM Proteoglycans (R-HSA-3000178) | 1/55 | 0,187 | LAMC1 |
| RHO GTPase Effectors (R-HSA-195258) | 2/269 | 0,189 | TAOK1,YWHAG |
| SCF(Skp2)-mediated Degradation Of P27/P21 (R-HSA-187577) | 1/59 | 0,197 | CCND1 |
| Formation Of beta-catenin:TCF Transactivating Complex (R-HSA-201722) | 1/60 | 0,198 | KMT2D |
| RHO GTPases Activate PKNs (R-HSA-5625740) | 1/62 | 0,203 | YWHAG |
| Signaling By MET (R-HSA-6806834) | 1/63 | 0,204 | LAMC1 |
| Extracellular Matrix Organization (R-HSA-1474244) | 2/291 | 0,207 | LAMC1,THBS1 |
| FOXO-mediated Transcription (R-HSA-9614085) | 1/65 | 0,207 | YWHAG |
| Integrin Cell Surface Interactions (R-HSA-216083) | 1/66 | 0,209 | THBS1 |
| Loss Of Nlp From Mitotic Centrosomes (R-HSA-380259) | 1/69 | 0,214 | YWHAG |
| Diseases Associated With O-glycosylation Of Proteins (R-HSA-3906995) | 1/69 | 0,214 | THBS1 |
| Plasma Lipoprotein Assembly, Remodeling, And Clearance (R-HSA-174824) | 1/71 | 0,217 | CLTC |
| trans-Golgi Network Vesicle Budding (R-HSA-199992) | 1/71 | 0,217 | CLTC |
| AURKA Activation By TPX2 (R-HSA-8854518) | 1/72 | 0,218 | YWHAG |
| ISG15 Antiviral Mechanism (R-HSA-1169408) | 1/75 | 0,225 | EIF4G2 |
| Infectious Disease (R-HSA-5663205) | 4/961 | 0,225 | LARP1,CLTC,G3BP1,YWHAG |
| Recruitment Of NuMA To Mitotic Centrosomes (R-HSA-380320) | 1/80 | 0,233 | YWHAG |
| Signaling By Rho GTPases (R-HSA-194315) | 3/644 | 0,233 | TAOK1,CLTC,YWHAG |
| Centrosome Maturation (R-HSA-380287) | 1/81 | 0,233 | YWHAG |
| Senescence-Associated Secretory Phenotype (SASP) (R-HSA-2559582) | 1/81 | 0,233 | CDK6 |
| Antiviral Mechanism By IFN-stimulated Genes (R-HSA-1169410) | 1/83 | 0,237 | EIF4G2 |
| Signaling By Rho GTPases, Miro GTPases And RHOBTB3 (R-HSA-9716542) | 3/660 | 0,242 | TAOK1,CLTC,YWHAG |
| Regulation Of PLK1 Activity At G2/M Transition (R-HSA-2565942) | 1/87 | 0,244 | YWHAG |
| PCP/CE Pathway (R-HSA-4086400) | 1/88 | 0,245 | CLTC |
| Regulation Of TP53 Activity Thru Phosphorylation (R-HSA-6804756) | 1/90 | 0,248 | TP53INP1 |
| Activation Of HOX Genes During Differentiation (R-HSA-5619507) | 1/91 | 0,248 | KMT2D |
| EPH-Ephrin Signaling (R-HSA-2682334) | 1/91 | 0,248 | CLTC |
| Unattached Kinetochores Signal Amplification Via A MAD2 Inhibitory Signal (R-HSA-141444) | 1/93 | 0,251 | TAOK1 |
| Transcriptional Regulation By RUNX3 (R-HSA-8878159) | 1/95 | 0,254 | CCND1 |
| Anchoring Of Basal Body To Plasma Membrane (R-HSA-5620912) | 1/97 | 0,256 | YWHAG |
| EML4 And NUDC In Mitotic Spindle Formation (R-HSA-9648025) | 1/97 | 0,256 | TAOK1 |
| Cytokine Signaling In Immune System (R-HSA-1280215) | 3/702 | 0,257 | CCND1,BRWD1,EIF4G2 |
| M Phase (R-HSA-68886) | 2/380 | 0,263 | TAOK1,YWHAG |
| Transport Of Inorganic Cations/Anions And Amino Acids/Oligopeptides (R-HSA-425393) | 1/104 | 0,264 | SLC20A1 |
| Cargo Recognition For Clathrin-Mediated Endocytosis (R-HSA-8856825) | 1/104 | 0,264 | CLTC |
| MHC Class II Antigen Presentation (R-HSA-2132295) | 1/104 | 0,264 | CLTC |
| Post-translational Protein Phosphorylation (R-HSA-8957275) | 1/106 | 0,264 | LAMC1 |
| Resolution Of Sister Chromatid Cohesion (R-HSA-2500257) | 1/106 | 0,264 | TAOK1 |
| O-linked Glycosylation (R-HSA-5173105) | 1/107 | 0,264 | THBS1 |
| Interleukin-4 And Interleukin-13 Signaling (R-HSA-6785807) | 1/107 | 0,264 | CCND1 |
| Degradation Of Extracellular Matrix (R-HSA-1474228) | 1/109 | 0,267 | LAMC1 |
| Mitotic Spindle Checkpoint (R-HSA-69618) | 1/110 | 0,268 | TAOK1 |
| Signaling By NTRK1 (TRKA) (R-HSA-187037) | 1/114 | 0,275 | CLTC |
| Epigenetic Regulation Of Gene Expression (R-HSA-212165) | 1/116 | 0,277 | TET3 |
| Transcriptional Regulation By RUNX2 (R-HSA-8878166) | 1/119 | 0,279 | CCND1 |
| RHO GTPases Activate Formins (R-HSA-5663220) | 1/119 | 0,279 | TAOK1 |
| Signaling By TGFB Family Members (R-HSA-9006936) | 1/119 | 0,279 | BMPR2 |
| Regulation Of IGF Transport And Uptake By IGFBPs (R-HSA-381426) | 1/123 | 0,285 | LAMC1 |
| Platelet Degranulation (R-HSA-114608) | 1/125 | 0,288 | THBS1 |
| Response To Elevated Platelet Cytosolic Ca2+ (R-HSA-76005) | 1/130 | 0,296 | THBS1 |
| Signaling By NTRKs (R-HSA-166520) | 1/132 | 0,298 | CLTC |
| Factors Involved In Megakaryocyte Development And Platelet Production (R-HSA-983231) | 1/136 | 0,303 | WEE1 |
| Signaling By Interleukins (R-HSA-449147) | 2/453 | 0,303 | CCND1,BRWD1 |
| Clathrin-mediated Endocytosis (R-HSA-8856828) | 1/142 | 0,312 | CLTC |
| Diseases Of Glycosylation (R-HSA-3781865) | 1/143 | 0,312 | THBS1 |
| Influenza Infection (R-HSA-168255) | 1/157 | 0,335 | CLTC |
| Regulation Of TP53 Activity (R-HSA-5633007) | 1/157 | 0,335 | TP53INP1 |
| Axon Guidance (R-HSA-422475) | 2/519 | 0,354 | CLTC,LAMC1 |
| Separation Of Sister Chromatids (R-HSA-2467813) | 1/170 | 0,354 | TAOK1 |
| mRNA Splicing - Major Pathway (R-HSA-72163) | 1/181 | 0,371 | SRRM2 |
| Nervous System Development (R-HSA-9675108) | 2/545 | 0,373 | CLTC,LAMC1 |
| Cilium Assembly (R-HSA-5617833) | 1/186 | 0,375 | YWHAG |
| mRNA Splicing (R-HSA-72172) | 1/189 | 0,378 | SRRM2 |
| TCF Dependent Signaling In Response To WNT (R-HSA-201681) | 1/198 | 0,390 | KMT2D |
| Interferon Signaling (R-HSA-913531) | 1/200 | 0,391 | EIF4G2 |
| Hemostasis (R-HSA-109582) | 2/576 | 0,391 | WEE1,THBS1 |
| Membrane Trafficking (R-HSA-199991) | 2/599 | 0,409 | CLTC,YWHAG |
| Mitotic Anaphase (R-HSA-68882) | 1/232 | 0,433 | TAOK1 |
| Mitotic Metaphase And Anaphase (R-HSA-2555396) | 1/233 | 0,433 | TAOK1 |
| Vesicle-mediated Transport (R-HSA-5653656) | 2/637 | 0,434 | CLTC,YWHAG |
| Immune System (R-HSA-168256) | 5/1943 | 0,437 | DDX3X,CCND1,CLTC,BRWD1,EIF4G2 |
| Processing Of Capped Intron-Containing Pre-mRNA (R-HSA-72203) | 1/242 | 0,437 | SRRM2 |
| Developmental Biology (R-HSA-1266738) | 3/1073 | 0,437 | KMT2D,CLTC,LAMC1 |
| SLC-mediated Transmembrane Transport (R-HSA-425407) | 1/247 | 0,439 | SLC20A1 |
| Diseases Of Metabolism (R-HSA-5668914) | 1/247 | 0,439 | THBS1 |
| Platelet Activation, Signaling And Aggregation (R-HSA-76002) | 1/254 | 0,446 | THBS1 |
| Organelle Biogenesis And Maintenance (R-HSA-1852241) | 1/275 | 0,471 | YWHAG |
| Transport Of Small Molecules (R-HSA-382551) | 2/706 | 0,471 | SLC20A1,CLTC |
| Deubiquitination (R-HSA-5688426) | 1/279 | 0,471 | YOD1 |
| Post-translational Protein Modification (R-HSA-597592) | 3/1383 | 0,599 | YOD1,LAMC1,THBS1 |
| Diseases Of Signal Transduction By Growth Factor Receptors And Second Messengers (R-HSA-5663202) | 1/424 | 0,620 | CLTC |
| RHO GTPase Cycle (R-HSA-9012999) | 1/441 | 0,632 | CLTC |
| Neutrophil Degranulation (R-HSA-6798695) | 1/468 | 0,651 | DDX3X |
| Metabolism Of RNA (R-HSA-8953854) | 1/666 | 0,778 | SRRM2 |
| Metabolism Of Proteins (R-HSA-392499) | 3/1890 | 0,793 | YOD1,LAMC1,THBS1 |
| Adaptive Immune System (R-HSA-1280218) | 1/733 | 0,803 | CLTC |
| Innate Immune System (R-HSA-168249) | 1/1035 | 0,898 | DDX3X |
