# Supplementary figures and images for: In silico analysis of prognostic and diagnostic significance of target genes from prostate cancer cell lines derived exomicroRNAs
Source: Cancer Cell Int. 2023 Nov 17;23:275. doi: 10.1186/s12935-023-03123-1 (PMC10655318; doi:10.1186/s12935-023-03123-1)

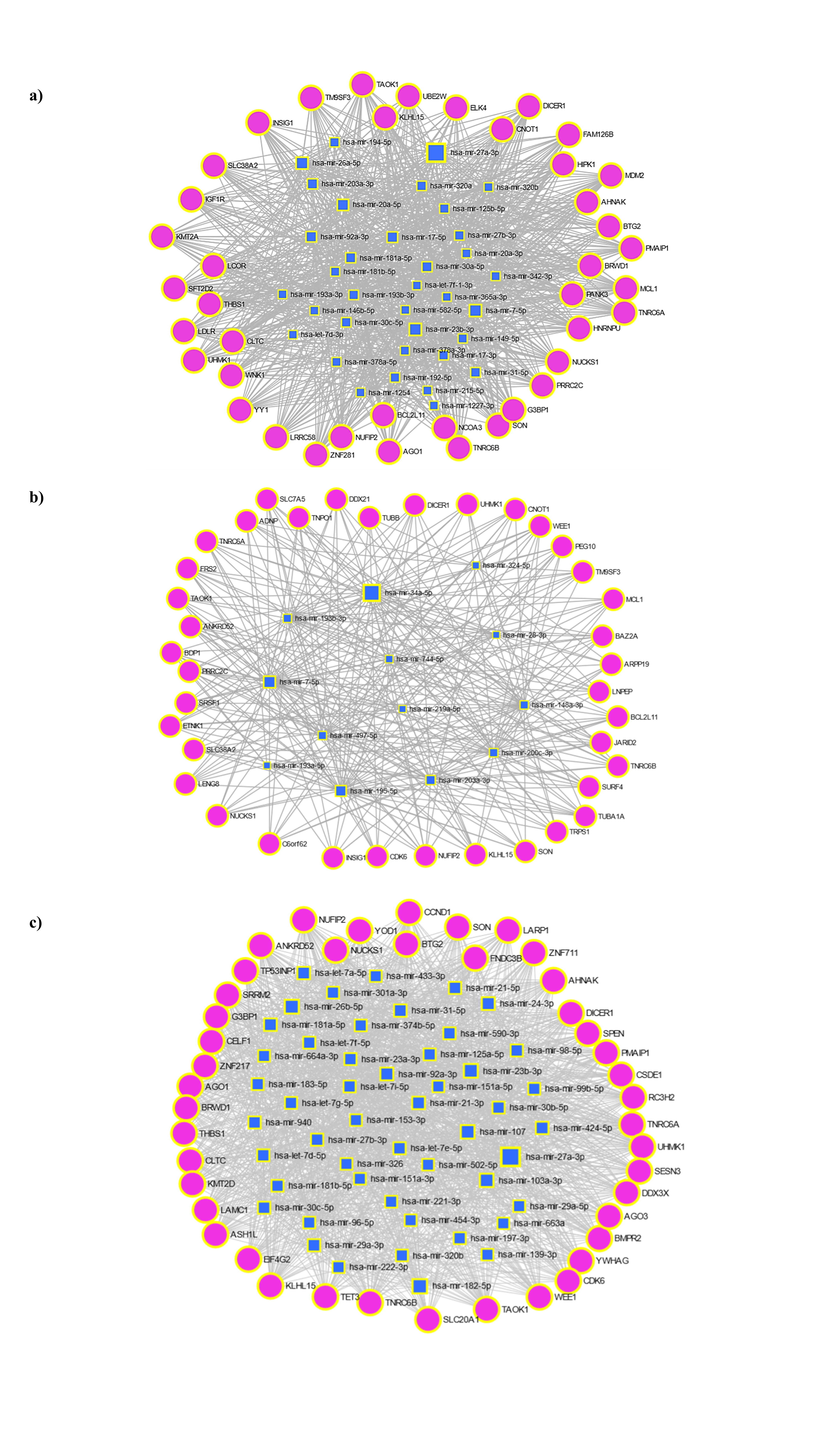

Supplement: Supplementary file 5 — Additional file 5: Figure S1. miRNeT Schematic representation of the interaction network of exomiRNAs with their targets-genes. a Network of exomiRNAs target genes regulated by at least 21/36 downregulated miRNAs in PCa cells vs RWPE-1 cells. b Network of exomiRNAs target genes regulated by at least 8/14 exomiRNAs downregulated miRNAs in PC-3 vs LNCaP cell line. c Network of exomiRNAs target genes regulated by at least 29/53 exomiRNAs downregulated miRNAs in PC-3 vs LNCaP cell line. Figure S2. Spearman correlation matrix. The Correlation map was plotted using significance levels for Spearman´s test performed with deregulated exomiRNAs and selected target genes. a Diagnostic study. b Prognostic study. Positive correlations are displayed in grading-blue and negative correlations in grading-red colour. Correlations with p-value ≥0.05 are considered insignificant and are left blank. Colour intensity and the size of the circle are proportional to the correlation coefficients. On the right side of the correlogram, the legend colour shows the correlation coefficients. Figure S3. a Boxplot showing mRNAs expression of CDK6, TNRC6B, AGO1, AGO3, and TNRC6A in prostate tumour tissue samples of patients stratified according to the ISUP-GG classification retrieved from the TCGA-PRAD database. Box plots show the median, quartiles, and extreme values. Different lettering over the boxes indicates statistical differences. Significant differences are established at p < 0.05. b Boxplot showing mRNA expression of CDK6, TNRC6B, and AGO1 in prostate tumour tissue samples of patients stratified according to affected lymph nodes split into N0 (no cancer in nearby lymph nodes) and N1 (cancer cells in 1 nearby lymph node) as stated in TCGA-PRAD database. Box plots show the median, quartiles, and extreme values. Different letter over the boxes indicates statistical differences. Significant differences are established at p < 0.05. [file 12935_2023_3123_MOESM5_ESM.zip › Figure S1/Fig_S1_REV1.tif]

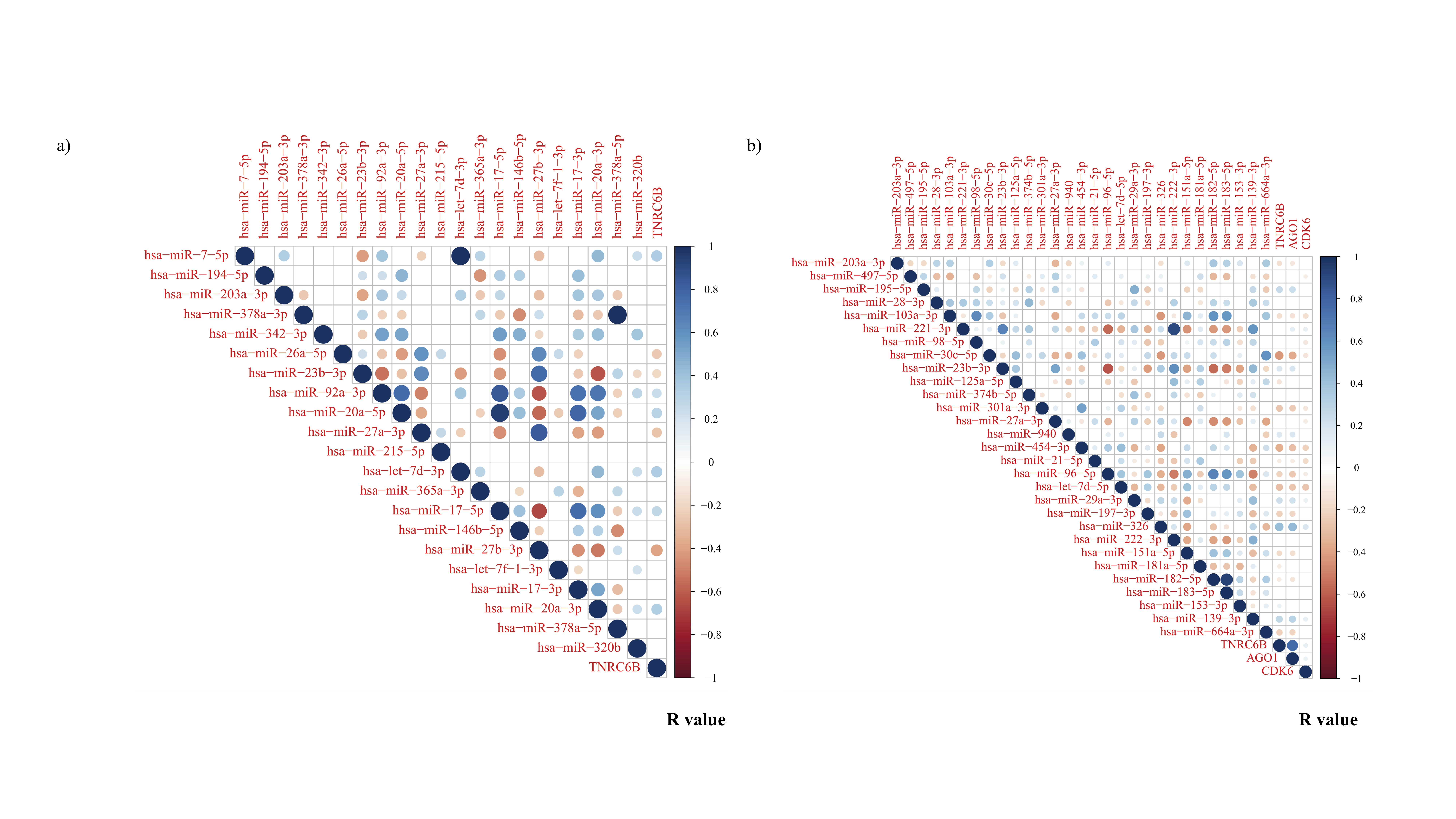

Supplement: Supplementary file 5 — Additional file 5: Figure S1. miRNeT Schematic representation of the interaction network of exomiRNAs with their targets-genes. a Network of exomiRNAs target genes regulated by at least 21/36 downregulated miRNAs in PCa cells vs RWPE-1 cells. b Network of exomiRNAs target genes regulated by at least 8/14 exomiRNAs downregulated miRNAs in PC-3 vs LNCaP cell line. c Network of exomiRNAs target genes regulated by at least 29/53 exomiRNAs downregulated miRNAs in PC-3 vs LNCaP cell line. Figure S2. Spearman correlation matrix. The Correlation map was plotted using significance levels for Spearman´s test performed with deregulated exomiRNAs and selected target genes. a Diagnostic study. b Prognostic study. Positive correlations are displayed in grading-blue and negative correlations in grading-red colour. Correlations with p-value ≥0.05 are considered insignificant and are left blank. Colour intensity and the size of the circle are proportional to the correlation coefficients. On the right side of the correlogram, the legend colour shows the correlation coefficients. Figure S3. a Boxplot showing mRNAs expression of CDK6, TNRC6B, AGO1, AGO3, and TNRC6A in prostate tumour tissue samples of patients stratified according to the ISUP-GG classification retrieved from the TCGA-PRAD database. Box plots show the median, quartiles, and extreme values. Different lettering over the boxes indicates statistical differences. Significant differences are established at p < 0.05. b Boxplot showing mRNA expression of CDK6, TNRC6B, and AGO1 in prostate tumour tissue samples of patients stratified according to affected lymph nodes split into N0 (no cancer in nearby lymph nodes) and N1 (cancer cells in 1 nearby lymph node) as stated in TCGA-PRAD database. Box plots show the median, quartiles, and extreme values. Different letter over the boxes indicates statistical differences. Significant differences are established at p < 0.05. [file 12935_2023_3123_MOESM5_ESM.zip › Figure S1/Fig_S2-REV1.tif]

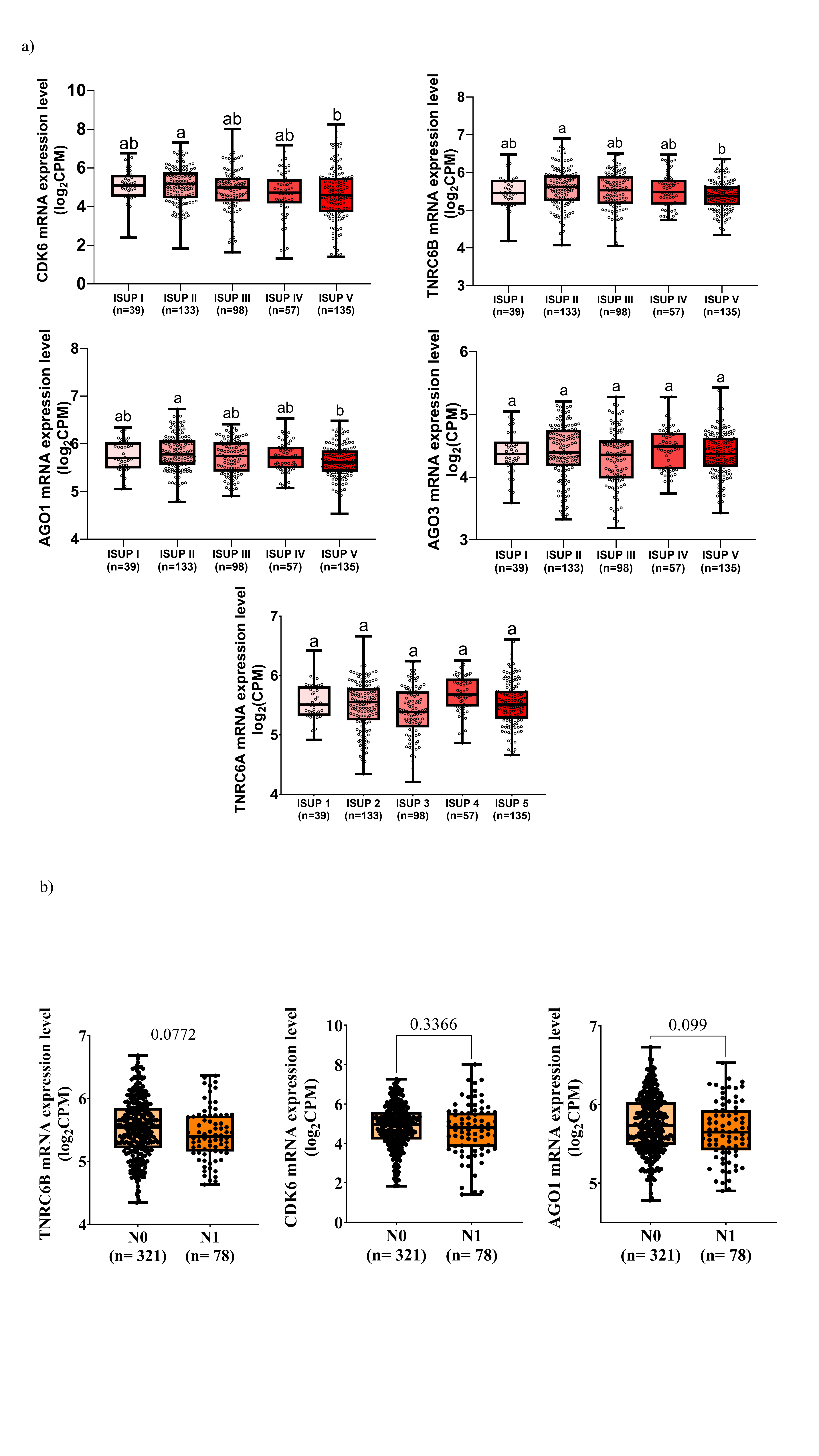

Supplement: Supplementary file 5 — Additional file 5: Figure S1. miRNeT Schematic representation of the interaction network of exomiRNAs with their targets-genes. a Network of exomiRNAs target genes regulated by at least 21/36 downregulated miRNAs in PCa cells vs RWPE-1 cells. b Network of exomiRNAs target genes regulated by at least 8/14 exomiRNAs downregulated miRNAs in PC-3 vs LNCaP cell line. c Network of exomiRNAs target genes regulated by at least 29/53 exomiRNAs downregulated miRNAs in PC-3 vs LNCaP cell line. Figure S2. Spearman correlation matrix. The Correlation map was plotted using significance levels for Spearman´s test performed with deregulated exomiRNAs and selected target genes. a Diagnostic study. b Prognostic study. Positive correlations are displayed in grading-blue and negative correlations in grading-red colour. Correlations with p-value ≥0.05 are considered insignificant and are left blank. Colour intensity and the size of the circle are proportional to the correlation coefficients. On the right side of the correlogram, the legend colour shows the correlation coefficients. Figure S3. a Boxplot showing mRNAs expression of CDK6, TNRC6B, AGO1, AGO3, and TNRC6A in prostate tumour tissue samples of patients stratified according to the ISUP-GG classification retrieved from the TCGA-PRAD database. Box plots show the median, quartiles, and extreme values. Different lettering over the boxes indicates statistical differences. Significant differences are established at p < 0.05. b Boxplot showing mRNA expression of CDK6, TNRC6B, and AGO1 in prostate tumour tissue samples of patients stratified according to affected lymph nodes split into N0 (no cancer in nearby lymph nodes) and N1 (cancer cells in 1 nearby lymph node) as stated in TCGA-PRAD database. Box plots show the median, quartiles, and extreme values. Different letter over the boxes indicates statistical differences. Significant differences are established at p < 0.05. [file 12935_2023_3123_MOESM5_ESM.zip › Figure S1/Fig_S3_REV1.tif]
