# Supplementary material for: In silico analysis of prognostic and diagnostic significance of target genes from prostate cancer cell lines derived exomicroRNAs
Source: Cancer Cell Int. 2023 Nov 17;23:275. doi: 10.1186/s12935-023-03123-1 (PMC10655318; doi:10.1186/s12935-023-03123-1)
Supplement: Supplementary file 6 — Additional file 6: Table S1. Patients’ characteristic used for diagnosis approach analysis. Table S2. Prediction of microRNA binding sites by miRWalk. Table S3. Patients’ characteristic used for prognosis approach analysis. [file 12935_2023_3123_MOESM6_ESM.zip › Tables/Table S2-MIRWAK.docx]

**Table S2.** miRNAs gene targets as show in miRWalk platform.

| miRNAs diagnosis | | | | | |
| --- | --- | --- | --- | --- | --- |
| miRNA | **Isoform TNRC6B** | **Binding Position** | **Start** | **End** | **Score** |
| hsa-miR-26a-5p | - | - | - | - | - |
| Hsa-miR-23b-3p | 1 | 3’-UTR | 16039 | 16078 | 1 |
|  | 2 | 3’-UTR | 15709 | 15748 | 1 |
|  | 3 | 3’-UTR | 13701 | 13740 | 1 |
| Hsa-miR-27a-3p | 1 | 3’-UTR | 11557 | 11580 | 0.92 |
|  |  | 3’-UTR | 6543 | 6557 | 0.92 |
|  | 2 | 3’-UTR | 11227 | 11250 | 0.92 |
|  |  | 3’-UTR | 6213 | 6227 | 0.92 |
|  | 3 | CDS | 835 | 857 | 1 |
|  |  | 3’-UTR | 9219 | 9242 | 0.92 |
| Hsa-miR-27b-3p | 1 | CDS | 5353 | 5374 | 0.92 |
|  |  | 3’-UTR | 6637 | 6659 | 0.84 |
|  | 2 | CDS | 5023 | 5044 | 0.92 |
|  |  | 3’-UTR | 6307 | 6329 | 0.85 |
|  | 3 | 3’-UTR | 3015 | 3036 | 0.92 |
|  |  | CDS | 4299 | 4321 | 0.85 |

|  | **miRNAs prognosis** | | | | | |
| --- | --- | --- | --- | --- | --- | --- |
| **Target Gen** | **miRNA** | **Isoform** | **Binding Position** | **Start** | **End** | **Score** |
| **TNRC6B** | **hsa-miR-103a-3p** | - | - | - | - | - |
|  | **hsa-miR-30c-5p** | 1 | 3’-UTR | 8981 | 9002 | 0.92 |
|  |  |  | CSD | 1466 | 1493 | 0.84 |
|  |  | 2 | 3’-UTR | 8651 | 48072 | 0.92 |
|  |  |  | CDS | 1466 | 1493 | 0.85 |
|  |  | 3 | 3’-UTR | 6643 | 6664 | 0.92 |
|  |  |  | 3’-UTR | 8844 | 8870 | 0.92 |
|  | **hsa-miR-301a-3p** | - | - | - | - | - |
|  | **hsa-miR-454-3p** | 2 | 3’-UTR | 15845 | 15866 | 0.88 |
|  | **hsa-miR-21-5p** | 2 | CDS | 1751 | 1772 | 1 |
|  | **hsa-miR-96-5p** | 2 | CDS | 409 | 442 | 0.95 |
|  |  | 3 | CDS | 591 | 624 | 0.95 |
|  | **hsa-let-7d-5p** | 1 | 3’-UTR | 7643 | 7667 | 0.95 |
|  |  |  | 3’-UTR | 7043 | 4060 | 0.85 |
|  |  | 2 | 3’-UTR | 7313 | 7337 | 0.95 |
|  |  |  | 3’-UTR | 6713 | 6730 | 0.85 |
|  |  | 3 | 3’-UTR | 5305 | 5329 | 0.95 |
|  |  |  | 3’-UTR | 4705 | 4722 | 0.85 |
| **CDK6** | **hsa-miR-103a-3p** | 1 | 3’-UTR | 7950 | 7973 | 0.85 |
|  |  |  | 3’-UTR | 1498 | 1521 | 0.85 |
|  |  | 2 | 3’-UTR | 7959 | 7982 | 0.85 |
|  |  |  | 3’-UTR | 1507 | 1530 | 0.85 |
|  | **hsa-miR-30c-5p** | - | - | - | - | - |
|  | **hsa-miR-301a-3p** | 1 | 3’-UTR | 4404 | 4432 | 0.85 |
|  |  |  | 3’-UTR | 9385 | 9413 | 0.85 |
|  |  | 2 | 3’-UTR | 4413 | 4441 | 0.85 |
|  |  |  | 3’-UTR | 9394 | 9422 | 0.85 |
|  | **hsa-miR-454-3p** | 1 | 3’-UTR | 3083 | 3106 | 0.85 |
|  |  | 2 | 3’-UTR | 3092 | 3115 | 0.85 |
|  | **hsa-miR-21-5p** | - | - | - | - | - |
|  | **hsa-miR-96-5p** | 1 | 3’-UTR | 9278 | 9306 | 0.92 |
|  |  | 2 | 3’-UTR | 9287 | 9315 | 0.92 |
|  | **hsa-let-7d-5p** | 1 | 3’-UTR | 2950 | 2975 | 0.85 |
|  |  |  | 3’-UTR | 2734 | 2752 | 1 |
|  |  |  | 3’-UTR | 10103 | 10128 | 1 |
|  |  | 2 | 3’-UTR | 2959 | 2984 | 0.85 |
|  |  |  | 3’-UTR | 2743 | 2761 | 1 |
|  |  |  | 3’-UTR | 10112 | 10137 | 1 |
| **AGO1** | **hsa-miR-103a-3p** | 1X | 3’-UTR | 12720 | 12738 | 1 |
|  |  |  | 3’-UTR | 3557 | 3587 | 0.85 |
|  |  |  | 3’-UTR | 3144 | 3167 | 1 |
|  |  | 2 | 3’-UTR | 12625 | 12643 | 1 |
|  |  |  | 3’-UTR | 3462 | 3492 | 0.85 |
|  |  |  | 3’-UTR | 3049 | 3072 | 1 |
|  | **hsa-miR-30c-5p** | 1x | 5’-UTR | 133 | 149 | 0.85 |
|  |  |  | CDS | 2879 | 2906 | 1 |
|  |  | 2 | 3’-UTR | 2784 | 2811 | 1 |
|  | **hsa-miR-301a-3p** | 1x | 3’-UTR | 13378 | 13394 | 0.85 |
|  |  | 2 | 3’-UTR | 13283 | 13299 | 0.85 |
|  | **hsa-miR-454-3p** | 1x | 3’-UTR | 7142 | 7169 | 1 |
|  |  | 2 | 3’-UTR | 7047 | 7074 | 1 |
|  | **hsa-miR-21-5p** | 1x | 3’-UTR | 4917 | 4937 | 0.85 |
|  |  | 2 | 3’-UTR | 4822 | 4842 | 0.85 |
|  | **hsa-miR-96-5p** | 1 | 5’-UTR | 132 | 158 | 0.85 |
|  |  |  | 3’-UTR | 12202 | 12233 | 0.92 |
|  |  | 1x | 3’-UTR | 12107 | 12138 | 0.92 |
|  |  | 2 | 3’-UTR | 12107 | 12138 | 0.92 |
|  | **hsa-let-7d-5p** | 1 | CDS | 1884 | 1903 | 0.87 |
|  |  |  | 3’-UTR | 4386 | 4404 | 1 |
|  |  |  | 3’-UTR | 5769 | 5790 | 0.85 |
|  |  | 1x | CDS | 1884 | 1903 | 1 |
|  |  |  | 3’-UTR | 4386 | 4404 | 1 |
|  |  |  | 3’-UTR | 5769 | 5790 | 0.92 |
|  |  | 2 | CDS | 1789 | 1808 | 1 |
|  |  |  | 3’-UTR | 4291 | 4309 | 1 |
|  |  |  | 3’-UTR | 5674 | 5695 | 0.92 |

CDS: coding sequence; 3’-UTR: 3' untranslated region; 5’-UTR: untranslated region.

The calculated score is generated by executing the TarPmiR algorithm for miRNA target site prediction. The closer the score is to 1, the greater is the confidence prediction: TNRCB6: Isoform 1 (NM_001162501.2), Isoform 2 (NM_015088.3) and Isoform 3 (NM_001024843.2); CK6: Isoform 1 ([NP_001250.1](https://www.ncbi.nlm.nih.gov/protein/NP_001250.1) ) and Isoform 2 ([NM_001145306.2](https://www.ncbi.nlm.nih.gov/nuccore/NM_001145306.2)); AGO1: Isoform 1(NM_012199.5), Isoform 1x (NM_001317122.2) and Isoform 2 (NM_001317123.2).
