# Supplementary material for: In silico analysis of prognostic and diagnostic significance of target genes from prostate cancer cell lines derived exomicroRNAs
Source: Cancer Cell Int. 2023 Nov 17;23:275. doi: 10.1186/s12935-023-03123-1 (PMC10655318; doi:10.1186/s12935-023-03123-1)
Supplement: Supplementary file 6 — Additional file 6: Table S1. Patients’ characteristic used for diagnosis approach analysis. Table S2. Prediction of microRNA binding sites by miRWalk. Table S3. Patients’ characteristic used for prognosis approach analysis. [file 12935_2023_3123_MOESM6_ESM.zip › Tables/Table_S1.docx]

**Table S1**

| **CANCER MIRNOME DIAGNOSTIC** | | |
| --- | --- | --- |
|  | | |
| **Patient's Characteristics** | Median (Range) | N |
| Age (years) | 60.29 (43.00, 72.00) | 52 |
| PSA (ng/ml) | 6.45 (4.60, 10.88) | 52 |
| **ISUP-GG** |  |  |
| Low Risk | Group I | 5 |
|  | Group II | 25 |
| High Risk | Group III | 15 |
|  | Group IV | 3 |
|  | Group V | 4 |
| **T pathological stage** | ≤T2a | 29 |
|  | T3,T4 | 23 |
| **N pathological stage** | NX | 5 |
|  | N0 | 46 |
|  | N1 | 1 |
| Abbreviations: ISUP-GG, International Society of Urological Pathology Gleason Grade groups based on the Gleason score as follows: (Gleason score ≤ 6 - Group I; 3 + 4 = 7 - Group II; 4 + 3 = 7 - Group III; 4 + 4 = 8 - Group IV; and 9-10-Group V); T stage, Tumor category; N stage, Node category. | | |
|  |  |  |
|  |  |  |
|  |  |  |
|  |  |  |
|  |  |  |
