# Supplementary material for: In silico analysis of prognostic and diagnostic significance of target genes from prostate cancer cell lines derived exomicroRNAs
Source: Cancer Cell Int. 2023 Nov 17;23:275. doi: 10.1186/s12935-023-03123-1 (PMC10655318; doi:10.1186/s12935-023-03123-1)
Supplement: Supplementary file 6 — Additional file 6: Table S1. Patients’ characteristic used for diagnosis approach analysis. Table S2. Prediction of microRNA binding sites by miRWalk. Table S3. Patients’ characteristic used for prognosis approach analysis. [file 12935_2023_3123_MOESM6_ESM.zip › Tables/Table_S3.docx]

**Table S3**

**CANCER MIRNOME PRONOSTIC**

| **Patient's Characteristics** | Median (Range) | N | *p*-value |  |
| --- | --- | --- | --- | --- |
| **Age (years)** | 61.00 (56.00, 66.00) | 465 |  |  |
| **PSA (ng/ml)** |  |  |  |  |
| Overall | 7.50 (5.10, 11.43) | 465 |  |  |
| Low Risk | 6.50 (4.90, 8.58) | 172 | < 0.001 |  |
| High Risk | 8.40 (5.35, 14.2) | 293 |  |  |
| **ISUP-GG** |  | N |  |  |
| Low Risk | Group I | 39 |  |  |
|  | Group II | 133 |  |  |
| High Risk | Group III | 98 |  |  |
|  | Group IV | 57 |  |  |
|  | Group V | 138 |  |  |
| **T pathological stage** | No data | 6 |  |  |
|  | T2a | 11 |  |  |
|  | T2b | 11 |  |  |
|  | T2c | 150 |  |  |
|  | T3a | 149 |  |  |
|  | T3b | 129 |  |  |
|  | T4 | 9 |  |  |
| **N pathological stage** | NX | 66 |  |  |
|  | N0 | 321 |  |  |
|  | N1 | 78 |  |  |
| Abbreviations: ISUP-GG, International Society of Urological Pathology Gleason Grade groups based on the Gleason score as follows: (Gleason score ≤ 6 - Group I; 3 + 4 = 7 - Group II; 4 + 3 = 7 - Group III; 4 + 4 = 8 - Group IV; and 9-10-Group V); T stage, Tumour category; N stage, Node category. | | | |  |
|  |  |  |  |  |
|  |  |  |  |  |
|  |  |  |  |  |
|  |  |  |  |  |
|  |  |  |  |  |
